# Preliminary report on harmonization of features extraction process using the ComBat tool in the multi-center “Blue Sky Radiomics” study on stage III unresectable NSCLC

**DOI:** 10.1186/s13244-022-01171-1

**Published:** 2022-03-07

**Authors:** Raffaella Fiamma Cabini, Francesca Brero, Andrea Lancia, Chiara Stelitano, Olga Oneta, Elena Ballante, Emanuela Puppo, Manuel Mariani, Emanuele Alì, Valentina Bartolomeo, Marianna Montesano, Elisa Merizzoli, Diana Aluia, Francesco Agustoni, Giulia Maria Stella, Roger Sun, Linda Bianchini, Eric Deutsch, Silvia Figini, Chandra Bortolotto, Lorenzo Preda, Alessandro Lascialfari, Andrea Riccardo Filippi

**Affiliations:** 1 Department of Mathematics, Pavia University, Pavia, Italy; 2 National Institute for Nuclear Physics (INFN), Pavia Division, Pavia, Italy; 3 Department of Physics, Pavia University, Pavia, Italy; 4 Radiation Oncology, Fondazione IRCCS Policlinico San Matteo, Pavia, Italy; 5 Radiology, Fondazione IRCCS Policlinico San Matteo, Pavia, Italy; 6 Department of Clinical Surgical, Diagnostic and Pediatric Sciences, Pavia University, Pavia, Italy; 7 IRCCS Mondino, Pavia, Italy; 8 Radiotherapy, Perugia University, Perugia, Italy; 9 Medical Oncology, Fondazione IRCCS Policlinico San Matteo, Pavia, Italy; 10 Respiratory Diseases, Fondazione IRCCS Policlinico San Matteo, Pavia, Italy; 11 Insititut Gustave Roussy, Villejuif, Paris, France; 12 Department of Social and Political Science, Pavia University, Pavia, Italy

**Keywords:** Robustness, Harmonization, ComBat, Radiomic features, NSCLC

## Abstract

**Background and purpose:**

In the retrospective-prospective multi-center "Blue Sky Radiomics” study (NCT04364776), we plan to test a pre-defined radiomic signature in a series of stage III unresectable NSCLC patients undergoing chemoradiotherapy and maintenance immunotherapy. As a necessary preliminary step, we explore the influence of different image-acquisition parameters on radiomic features’ reproducibility and apply methods for harmonization.

**Material and methods:**

We identified the primary lung tumor on two computed tomography (CT) series for each patient, acquired before and after chemoradiation with i.v. contrast medium and with different scanners. Tumor segmentation was performed by two oncological imaging specialists (thoracic radiologist and radio-oncologist) using the Oncentra Masterplan® software. We extracted 42 radiomic features from the specific ROIs (LIFEx). To assess the impact of different acquisition parameters on features extraction, we used the Combat tool with nonparametric adjustment and the longitudinal version (LongComBat).

**Results:**

We defined 14 CT acquisition protocols for the harmonization process. Before harmonization, 76% of the features were significantly influenced by these protocols. After, all extracted features resulted in being independent of the acquisition parameters. In contrast, 5% of the LongComBat harmonized features still depended on acquisition protocols.

**Conclusions:**

We reduced the impact of different CT acquisition protocols on radiomic features extraction in a group of patients enrolled in a radiomic study on stage III NSCLC. The harmonization process appears essential for the quality of radiomic data and for their reproducibility.

*ClinicalTrials.gov Identifier*: NCT04364776, First Posted:April 28, 2020, Actual Study Start Date: April 15, 2020, https://clinicaltrials.gov/ct2/show/NCT04364776.

**Supplementary Information:**

The online version contains supplementary material available at 10.1186/s13244-022-01171-1.

## Key points

- Radiomics may allow the translation of CT scan images into quantitative data to provide crucial information on intrinsic tumor heterogeneity, cancer behavior, and eventually response to therapy.
- One of the main limitations of the radiomics workflow, especially for its wide reproducibility in multi-center studies, is the variability of CT scanner models, acquisition protocols, and reconstruction algorithms.
- The harmonization of CT scan images through the ComBat and longComBat algorithms may reduce the impact of different acquisition protocols on radiomic features extraction.

## Introduction

Non-Small Cell Lung Cancer (NSCLC) accounts for about 75–80% of all diagnoses of lung cancer, and approximately one-third of these cases correspond to locally advanced stages (IIIA-C) [1]. The current therapeutic standard is curative intent concurrent or sequential chemoradiotherapy (CRT), with a platinum-based doublet and once-daily radiation dose up to 60 Gy, followed by the anti-PDL-1 monoclonal antibody Durvalumab in responding patients. This approach leads to a median overall survival (OS) of 47.5 months and progression-free survival (PFS) of 16.9 months, with 47.5% of patients alive at five years [2, 3]. However, despite these clinical improvements, 2/3 of patients still progress, most of them in the thorax, within irradiated volumes. The mechanisms underlying this resistance are not fully known.

Furthermore, there are no reliable biomarkers currently available to predict which patients best respond to immunotherapy [4, 5], even if preliminary data on circulating cell-free DNA are encouraging [6]. Therefore, finding and validating a more accurate way to better select patients who can benefit from immunotherapy would be of utmost importance. In this scenario, the opportunity to generate image-based biomarkers using radiomics has aroused great interest [7–9]. Such an approach is based on extracting several quantitative variables, known as radiomics features, and using them for building predictive models based on machine-learning classifiers [9]. However, one of the main limitations of the radiomics workflow is the variability of scanner models, acquisition protocols, and reconstruction algorithms. Such variations can significantly impact radiomic features' stability, especially for heterogeneous imaging data sets from radiomics multi-center studies, impairing the robustness of predictive models [10, 11].

Over the last few years, different research groups proposed methods to overcome these obstacles through a harmonization process. Da-Ano et al. [12] studied the use of different modified ComBat algorithms [13, 14], comparing the methods in a multi-center study involving two datasets of locally advanced cervical cancer patients from 3 centers, with magnetic resonance imaging and positron emission tomography imaging. They demonstrated that the quality of radiomic models increased with the use of the improved ComBat method. Masson et al. [15] evaluated the use of ComBat as a radiomic feature harmonization method in patients with laryngeal cancer from five different centers, showing an increased predictive value. Mahon et al. [16] also used the ComBat method, demonstrating that it can be used in multi-institutional studies to harmonize radiomic features extracted from images acquired using different CT protocols in patients with lung tumors.

In this preliminary report, we explore the influence of image acquisition parameters on radiomic features extraction reproducibility, considering both differences between scanners and acquisition protocols, and we propose harmonization methods to minimize the data analysis variability. The main element of novelty is the application of a modification of the ComBat algorithm, called LongCombat [17], to radiomic features. Moreover, we compare the original and the modified longitudinal ComBat algorithms. Finally, by applying the proposed harmonization process, we aim to strengthen the prediction model that will hopefully be obtained by the final analysis of the Blue Sky dataset. The study included 23 patients, who underwent specific treatment (chemoradiotherapy and maintenance immunotherapy); for this reason, no public datasets were included, as they would not have been comparable to the specific cohort of patients enrolled in the Blue Sky Radiomics study. Moreover, the use of the Blue Sky dataset, which is part of an observational clinical trial [18], reduces further variability due to the different operators contouring the ROIs, and less control over a series of processes (related to image acquisition, contouring and clinical selection).

## Materials and methods

### Data

The study included 23 patients. The primary tumor was identified and delineated on two CT series with i.v. contrast medium, performed before and after CRT. The contouring process was centralized and performed by oncological imaging experts using Masterplan Oncentra® software (https://medicaldevices.icij.org/devices/che-oncentra-external-beam). The images come from 10 different medical centers and are characterized by different acquisition and reconstruction protocols. The details are shown in Table 1.

**Table 1:** Summary of imaging acquisition parameters and values

Acquisition parameters	Value
Scanner manufacturer	Philips, Siemens, Toshiba, GE
kVp	100, 120, 130, 140
Convolution kernel	B^a^,B10f^b^,B20f^b^,B30f^b^,B31f^b^, B31s^b^,B40f^b^, FC08^c^, FC17^c^, STANDARD^d^
Exposure time (ms)	350, 500, 600, 698, 1000
Tube current (mA)	From 56 to 581
Contrast agents	Iobitridol, Iomeprol
Slice thickness (mm)	0.5, 1, 1.25, 1.5, 2, 2.5, 3

^a^Philips; ^b^Siemens; ^c^Toshiba; ^d^G.E; ^e^Non-ionic CA, market under the trade name Xenetix (Guerbert); ^f^monomeric and water-soluble non-ionic, injectable iodinated contrast medium solution (Bracco)

### Features extraction

Radiomic features extraction was performed using the LIFEx software v6.30 (Local Image Feature Extraction, IBSI standard-compliant [19]). A total of forty-two features were obtained with 3D extraction from each region of interest (ROI), corresponding to the tumor volume and secondary lesions. The ROI should include at least 64 voxels. The selected features include six categories: four shape features, six first-order statistics features, seven gray-level co-occurrence matrix (GLCM) features, eleven gray-level run-length matrix (GLRLM) features, three neighboring gray-level difference matrix (NGLDM) features, and eleven gray-level zone length matrix (GLZLM) features, as defined in [20]. In addition, we applied the following pre-processing steps before feature extraction to reduce variability between images: gray-levels absolute discretization and voxel resampling. The gray-level discretization was performed in the range [− 1000; 3000] Hounsfield Units (HU), with a bin number of 400 (bin-width 10 HU), while we resampled voxels to the fixed size of 1 mm × 1 mm × 1 mm.

### Harmonization

In order to reduce the influence of different CT acquisition protocols on the set of radiomic features, we used the ComBat harmonization tool [13, 14] and its version for longitudinal studies [17].

The ComBat harmonization technique belongs to the location and scale (L/S) adjustment methods which aim to eliminate the effects of batches by standardizing the means (location) and variances (scale) of each feature across batches. We chose the nonparametric setting of the model to avoid assumptions on the underlying probability distributions of the features and the parameters. Moreover, we did not include biological covariates, excluding the Bayesian setting, as the cohort of patients enrolled for the Blue Sky study is homogeneous. Thus, we do not have a significant biological feature that we would like to preserve from harmonization. By selecting these options, the algorithm computes a location- and-scale correction transformation for each feature separately, i.e., it adjusts the means and the variances of the distributions to reduce heterogeneity. We used the ComBat tool proposed in [21] and then adapted to multi-site imaging data in [13] (publicly available at https://github.com/Jfortin1/ComBatHarmonization), using the R software.

Moreover, we used a development of the ComBat tool, named longComBat, as defined in [17]. In longComBat, the original ComBat algorithm was adapted to longitudinal data when the independence requirement between statistical units was not satisfied. As far as we know, the application of this technique to radiomic features has not been studied yet. Unlike the cross-sectional ComBat tool, the longitudinal version is not yet provided with the nonparametric adjustment nor the possibility to exclude the Bayesian setting. We use the longCombat algorithm publicly available at https://github.com/jcbeer/longCombat, using the R software.

Further details about the algorithms are provided in Additional file 1: Appendix A.

### Statistical analyses

We ran all statistical analyses with RStudio (R Core Team, 2020; R Foundation for Statistical Computing, Vienna, Austria; https://www.R-project.org/); figures were produced using the package ggplot2 (H. Wickham; Springer-Verlag New York, 2016).

To assess the influence of the different acquisition parameters on the feature values, we performed the Kruskal–Wallis test before the ComBat harmonization. The level of statistical significance was set at a *p* value < 0.05 for all analyses. If the *p* value after the Kruskal–Wallis test is smaller than the significant threshold, this indicates that this feature's distribution had a statistical difference among groups; hence, the selected feature was affected by the considered acquisition parameter. The features were tested independently.

Once the most influential acquisition parameters were identified, we combined the significant parameters to harmonize the feature values across the batches. After the ComBat (both in cross-sectional and longitudinal case) compensation, the Kruskal–Wallis test was repeated to verify if the normalization was successful. If the *p* value of the test was greater than the threshold value, the feature distributions across the batches were correctly realigned.

## Results

We aim to tackle the variability in acquisition parameters using the ComBat tool and compare it with the longComBat, which explicitly considers the within-subject correlation inherent to longitudinal studies. Different acquisition parameters were explored: Scanner, kVp, Convolution Kernel, Contrast Agent, Exposure time. The X-ray tube flow (mA) was excluded from the harmonization, as modern CT scans work in dose modulation (or analog mA × s) by optimizing the dose in the patient's different regions, guaranteeing a high image "renormalized" quality. The flow is increased when the beam must cross regions with a high total attenuation coefficient. By reducing the flow, there is an increase in noise in the CT image, increasing the range of Hounsfield values. It has been observed that these variations have a more evident influence on inhomogeneous materials than in heterogeneous ones. Thus, the features extracted from heterogeneous subjects, like a patient's chest, are not dependent on the X-ray tube current [22].

We performed the statistical analysis and the cross-sectional harmonization considering both pre-CRT and post-CRT features. Through statistical testing, we identified kVp and Convolution Kernel as the most influential parameters. The scanner model resulted in 19 significant features of the 42 tested, the kVp in 21, the Convolution Kernel in 19, the Contrast Agent in 12, and the exposure time in 0. The scanner was excluded as strongly correlated with the Convolution Kernel, and Contrast Agent was excluded for having 50% of missing values. The exposure time does not have any influence on the features.

Basing on the combination of the parameters that show a significant association with the radiomic features (kVp, Convolution Kernel), we defined 15 CT acquisition protocols selected for the harmonization process, as described in Table 2. One image was excluded from the analysis (corresponding to protocol number 6) as for the application of the ComBat algorithm, a frequency of at least two for each protocol is necessary. Therefore, 14 protocols for statistical analysis and harmonization were considered. An integer numerical value, called batch, was associated with each possible combination of the convolutional kernel-kVp parameters.

**Table 2:** Summary of different imaging acquisition protocols necessary for the ComBat harmonization

Protocol	Convolution Kernel	kVp	Frequency
1	B	120	5
2	B10f	120	17
3	B20f	120	12
4	B30f	100	25
5	B30f	120	2
6	B30f	140	1
7	B31f	100	19
8	B31s	130	9
9	B31f	120	9
10	B40f	100	4
11	B40f	120	7
12	FC08	100	11
13	FC08	120	2
14	FC17	100	2
15	STANDARD	120	2

Frequency refers to the number of malignant lesions for each batch

The distribution of features' values across protocols was tested before the harmonization, and 76% resulted significantly influenced (*p* < 0.05). However, after harmonization, all extracted features were independent of the technical parameters of image acquisition (*p* > 0.05), showing that the ComBat method successfully eliminated the protocols' influence.

**Fig. 1:**
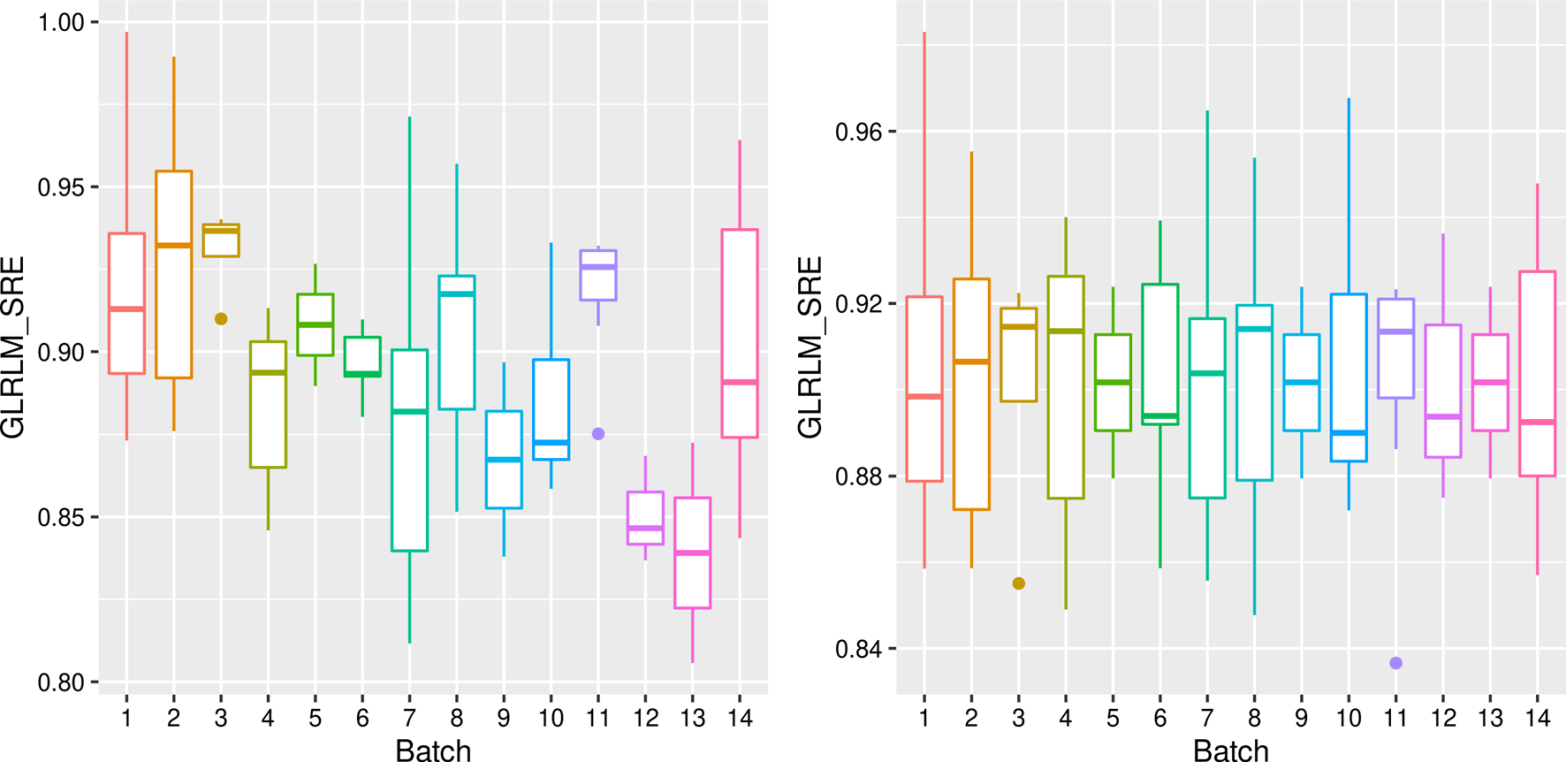
An example of feature harmonization for the GLRLM SRE feature. On the left is the box-plot for the feature distribution across batches (defined as protocols) before ComBat harmonization, and on the right is the Box-plot after the harmonization

An example of the harmonization effect is shown in Fig. 1, where we depict the dependence of the feature GLRLM Short Run Emphasis (GLRLM SRE) from the acquisition protocols before and after harmonization. Other examples are shown in the Supporting Information, where we represented the distributions of one exemplifying feature for each of the six categories described in “Features extraction” section.

**Fig. 2:**
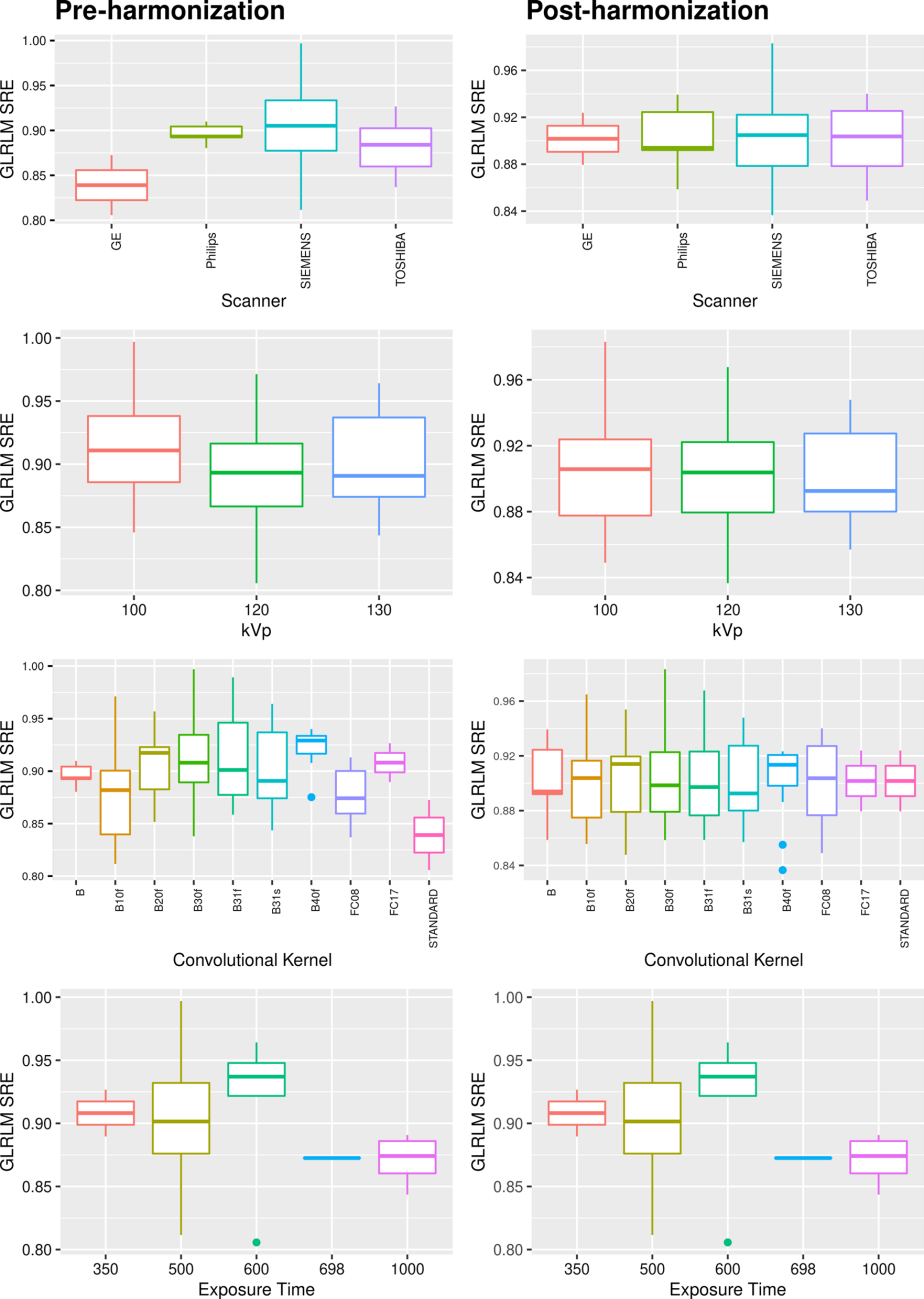
Statistical box-plots of GLRLM SRE feature distribution (pre-harmonization and post-harmonization with ComBat) across different image-acquisition parameters: Scanner, kVp, Convolutional Kernel, Exposure Time

We assessed the dependence of the feature values on the single acquisition parameters after the ComBat harmonization. Repeating the Kruskal–Wallis test, we obtained that 0 features of the 42 tested were influenced by the scanner manufacturer, 0 features were dependent on the kVp, 0 features were affected by the Convolution Kernel. These results confirm that the ComBat algorithm effectively removes the dependence of the radiomic features from the acquisition parameters. For illustrative purposes, the same feature GLRLM SRE is represented in Fig. 2, where the dependence from the single parameters is investigated.

The same 14 CT acquisition protocols were used for the harmonization process through the longComBat tool. In addition, to properly account for the dependence of repeated within-subject observations, the information about the time point of image acquisition (baseline or follow-up) was added.

**Fig. 3:**
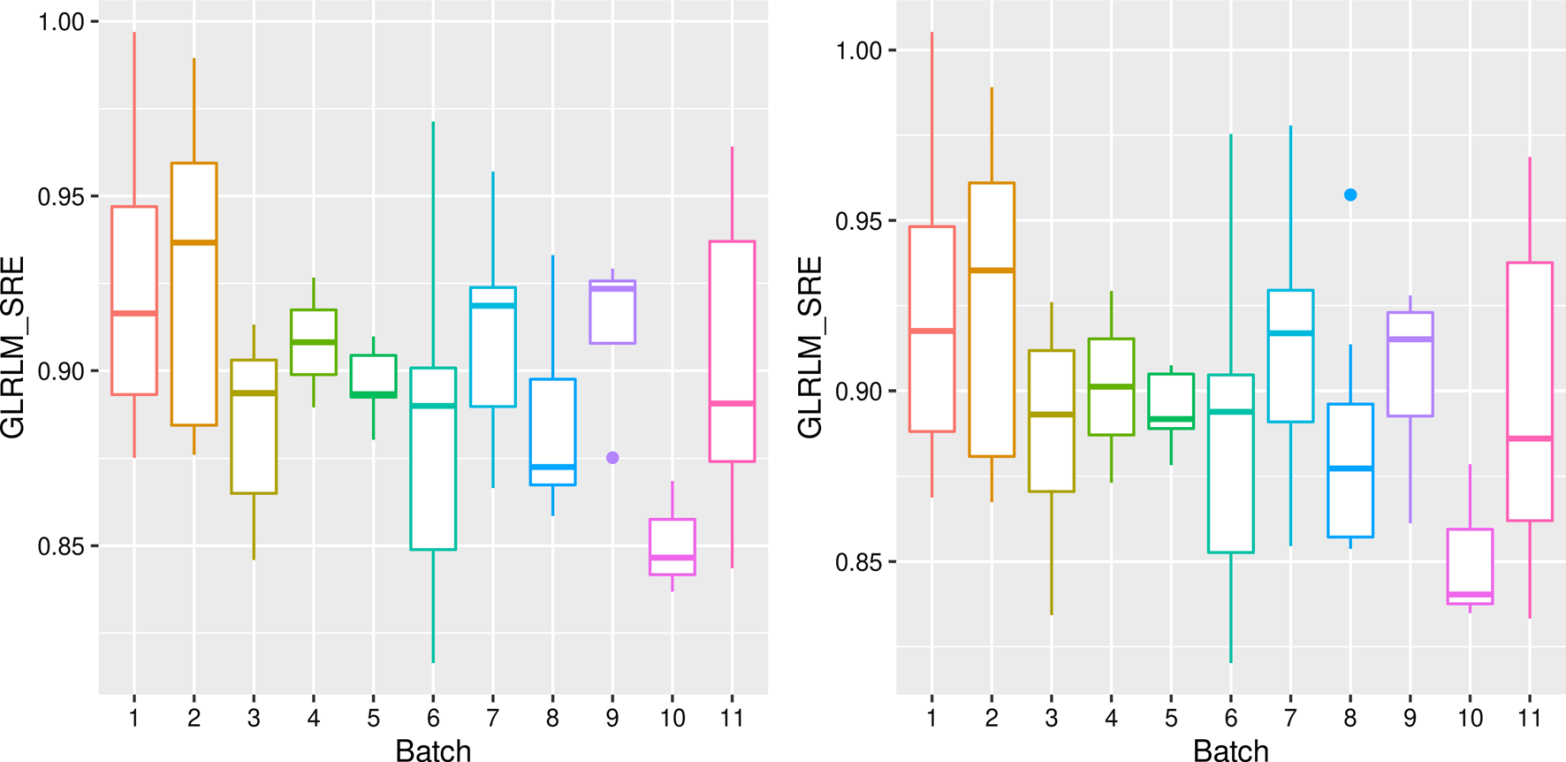
An example of feature harmonization for a sample feature, GLRLM SRE. On the left is the box-plot for the feature distribution across batches (defined as protocols) before longComBat harmonization, and on the right is the Box-plot after the harmonization

As the longitudinal algorithm requires at least one image per protocol and at least two-time points per patient, five subjects were removed from the analysis. The filtered dataset included images of 11 protocols: protocols 5, 6, 10, and 15 defined in Table 2 were eliminated. The tests performed before the harmonization show that these protocols significantly influenced 25 of the 42 radiomic features (59%). After the harmonization procedure, only two features (5%) significantly depended on the protocols. An example of the long-ComBat harmonization effect is shown in Fig. 3 (see Additional file 1 for other examples), where we depict the dependence of the GLRLM Short Run Emphasis (GLRLM SRE) feature from the acquisition protocols before and after harmonization.

**Fig. 4:**
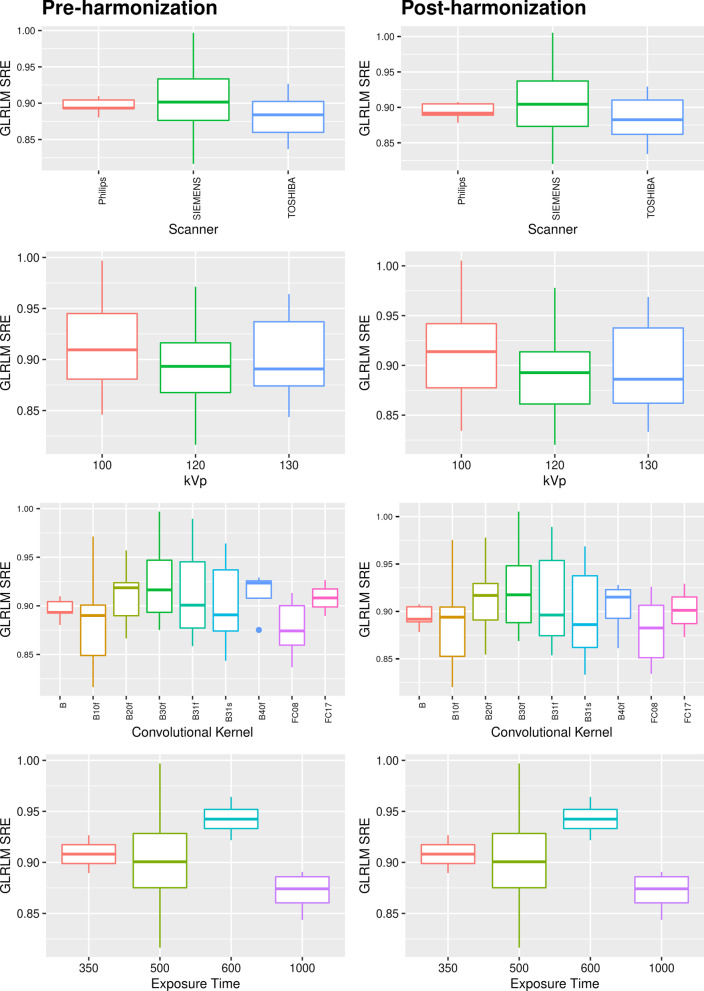
Statistical box-plots of GLRLM SRE feature distribution (pre- and post-harmonization with longComBat) across different image-acquisition parameters: Scanner, kVp, Convolutional Kernel, Exposure Time

Unlike the cross-sectional ComBat, the dependence of the feature values on single acquisition parameters after the longComBat harmonization was not entirely removed. For example, 4/42 features were dependent on the scanner manufacturer, five from the kVp, and two from the Convolution Kernel. For illustrative purposes, in Fig. 4, we represented the dependence of the GLRLM SRE feature from single parameters.

The harmonization process using cross-sectional ComBat was more robust, as the dependencies were entirely removed; the median values among protocols and parameters seem to be more homogeneous than those obtained with longCombat. The obvious advantage is a more substantial harmonization of the feature, while a possible drawback is the loss of information.

## Discussion

Images' intrinsic variability may largely influence Radiomic features. Images' inhomogeneity depends on different elements, such as scanners with different acquisition protocols and technical parameters. In order to address this issue, Lambin et al. [9] introduced the concept of radiomics quality score (RQS).

The use of this score allows assessing the quality of radiomic studies, analyzing each phase: data selection, medical imaging, feature extraction, exploratory analysis, and modeling. Regarding data selection, one of the main problems is the use of non-standardized acquisition protocols; according to RQS, this issue can be addressed by disclosing acquisition protocols in the radiological reports to reach a wide diffusion for reporting guidelines. The variability between different scanners could be managed using phantom studies. However, it is not easy to implement these solutions as many radiomic studies are retrospective, involving patients examined by different scanners, geographic regions, and times. The expected decay of the CT scanner tube may also lead to heterogeneity in image acquisition.

In the present study, we applied the ComBat algorithm for the statistical harmonization of radiomic features, and we compared it to its longitudinal version.

We tested the ComBat tool in different conditions from those already reported for two main aspects: (i) the comparison between the cross-sectional ComBat tool and the longitudinal version (longComBat) and (ii) the a-posteriori definition of harmonization batches from the combinations of kVp and Convolutional Kernel. We demonstrated that both harmonization algorithms adequately compensated the feature values by reducing the influence of the acquisition protocol. However, further works could include in the longComBat tool also the nonparametric setting and the non-Bayesian formulation. Concerning the second aspect, because of the small-sized dataset, we preferred to limit the number of acquisition parameters defining the harmonization protocols, including a pre-processing image phase which consisted of pixel normalization and gray-scale quantization. We were able to standardize images before feature extraction through this pre-processing step, and we avoided including the slice thickness and the pixel width among the harmonization parameters. However, this strategy can only be applied if the original CT images are available and not only the set of the radiomic features. We aimed to tackle the variability in acquisition parameters applying the ComBat tool and comparing it with the longComBat, which explicitly considers the within-subject correlation inherent to longitudinal studies. Both ComBat algorithms provide satisfactory results even for small datasets; indeed, both procedures decrease the features dependencies from the acquisition protocols below 5%.

As for the dependence from the single acquisition parameter, ComBat guarantees 100% harmonization, while longComBat reduces the number of dependencies to 12% at most. Our study wants to underline the usefulness of ComBat and, also, LongComBat. The ideal harmonization process to reduce the variation in radiomic features would be to harmonize the images before the acquisition, but it is clearly not always achievable, and this is the reason why harmonization methods like ComBat were developed. As already described in the literature cited above, the ComBat tool harmonizes radiomic features extracted from different imaging protocols. But thanks to picture archiving and communication systems (PACS), the original CT images are often available, so we have introduced a pre-processing step to standardize images before feature extraction with LIFEx, obtaining good results and getting closer to the ideal method of standardization of the features. The hope is that this study will stimulate research and development of different ComBat algorithms.

The main limitation of our study is the small number of patients, the object of this preliminary analysis, enrolled so far. At the same time, larger datasets would help make more robust conclusions about the harmonization process in the future.

In conclusion, our results showed the ComBat tool's ability to harmonize CT images for radiomic features extraction in lung cancer CT scans. These results will be beneficial for increasing the quality of the radiomic features extraction procedure and the analysis in the retrospective-prospective multi-center Blue Sky Radiomics study on stage III NSCLC.

## Supplementary Information

**Additional file 1.** Distributions of DISCRETIZED_HISTO_Skewness, GLCM_Correlation, GLZLM_ZP, NGLDM_Coarseness and SHAPE_Sphericity features across batches and image-acquisition parameters before and after the harmonization procedure (ComBat and Long ComBat).

## Data Availability

Data sharing does not apply to this article as no datasets were generated or analyzed during the current study.

## Abbreviations

CRT: Chemoradiotherapy
CT: Computed Tomography
GLCM: Gray-level co-occurrence matrix
GLRLM SRE: GLRLM Short Run Emphasis
GLRLM: Gray-level run-length matrix
GLZLM: Gray-level zone length matrix
HU: Hounsfield Units
NGLDM: Neighboring gray-level difference matrix
NSCLC: Non-Small Cell Lung Cancer
OS: Overall Survival
PACS: Picture archiving and communication system
PFS: Progression-free survival
ROI: Region Of Interest
RQS: Radiomics quality score
