# Supplementary material for: Preliminary report on harmonization of features extraction process using the ComBat tool in the multi-center “Blue Sky Radiomics” study on stage III unresectable NSCLC
Source: Insights Imaging. 2022 Mar 7;13:38. doi: 10.1186/s13244-022-01171-1 (PMC8901939; doi:10.1186/s13244-022-01171-1)
Supplement: Supplementary file 1 — Additional file 1. Distributions of DISCRETIZED_HISTO_Skewness, GLCM_Correlation, GLZLM_ZP, NGLDM_Coarseness and SHAPE_Sphericity features across batches and image-acquisition parameters before and after the harmonization procedure (ComBat and Long ComBat). [file 13244_2022_1171_MOESM1_ESM.docx]

**ELECTRONIC SUPPLEMENTARY MATERIAL**

***Preliminary report on harmonization of features extraction process using the ComBat tool in the multi-center “Blue Sky Radiomics” study on stage III unresectable NSCLC.***

- **Supplementary Figures S1 (A,B), S2 (A,B), S3 (A,B), S4 (A,B), S5 (A,B), S6 (A,B), S7 (A,B), S8 (A,B), S9 (A,B), S10 (A,B)**
- **Appendix A**

**With the ComBat tool:**

Figure S1 (A,B): DISCRETIZED_HISTO_Skewness,

Figure S2 (A,B): GLCM_Correlation

Figure S3 (A,B): GLZLM_ZP

Figure S4 (A,B): NGLDM_Coarseness

Figure S5 (A,B): SHAPE_Sphericity

**With the longComBat tool:**

Figure S6 (A,B): DISCRETIZED_HISTO_Skewness,

Figure S7 (A,B): GLCM_Correlation

Figure S8 (A,B): GLZLM_ZP

Figure S9 (A,B): NGLDM_Coarseness

Figure S10 (A,B): SHAPE_Sphericity

**A:** Box-plot distribution across batches for the 14 protocols: on the left before harmonization and on the right after harmonization.

**B:** Statistical box-plots for the 14 protocols: on the left pre-harmonization and on the right post-harmonization, across different image-acquisition parameters: Scanner, kVp, Convolutional Kernel, Exposure Time.

**Figure S1A**


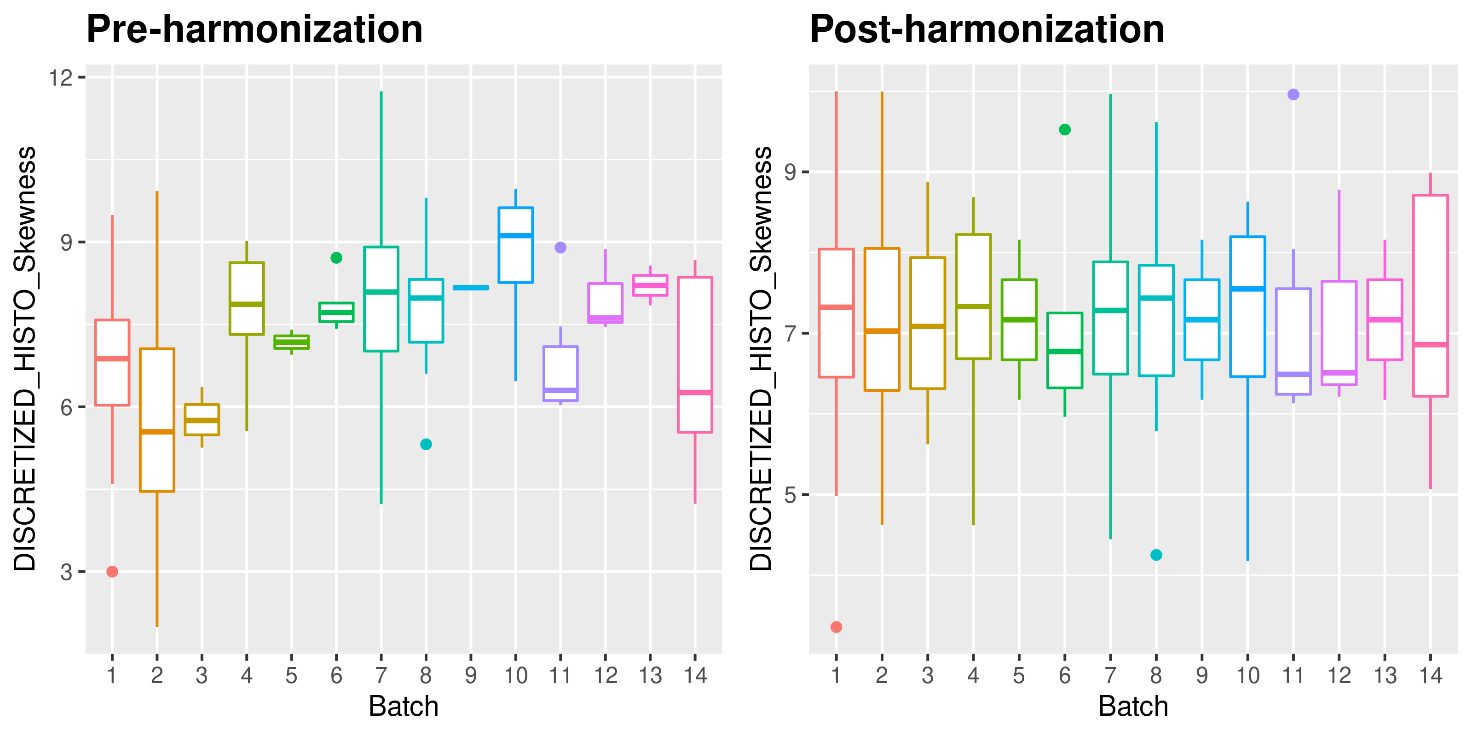


**Figure S1B**
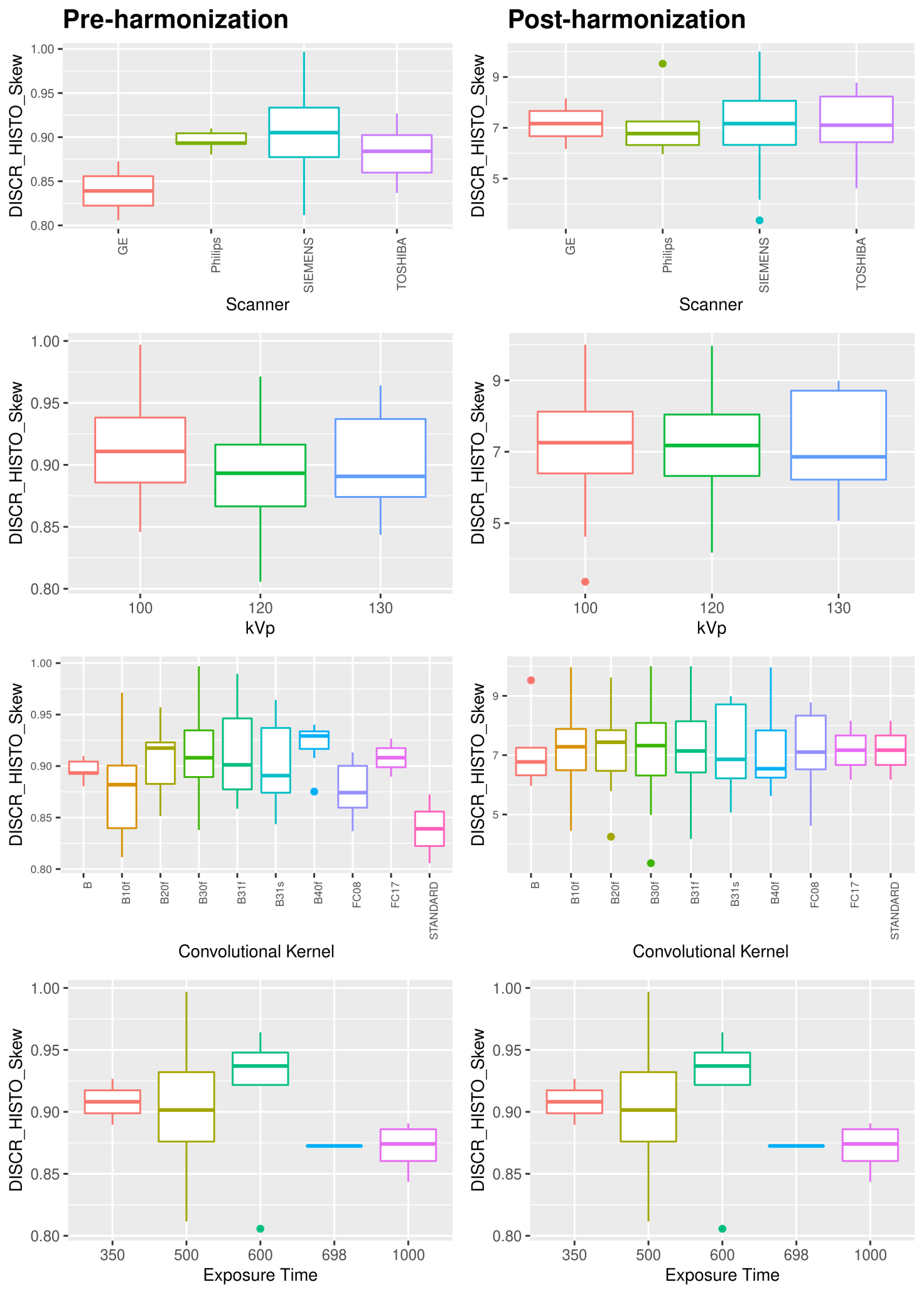


**Figure S2A**


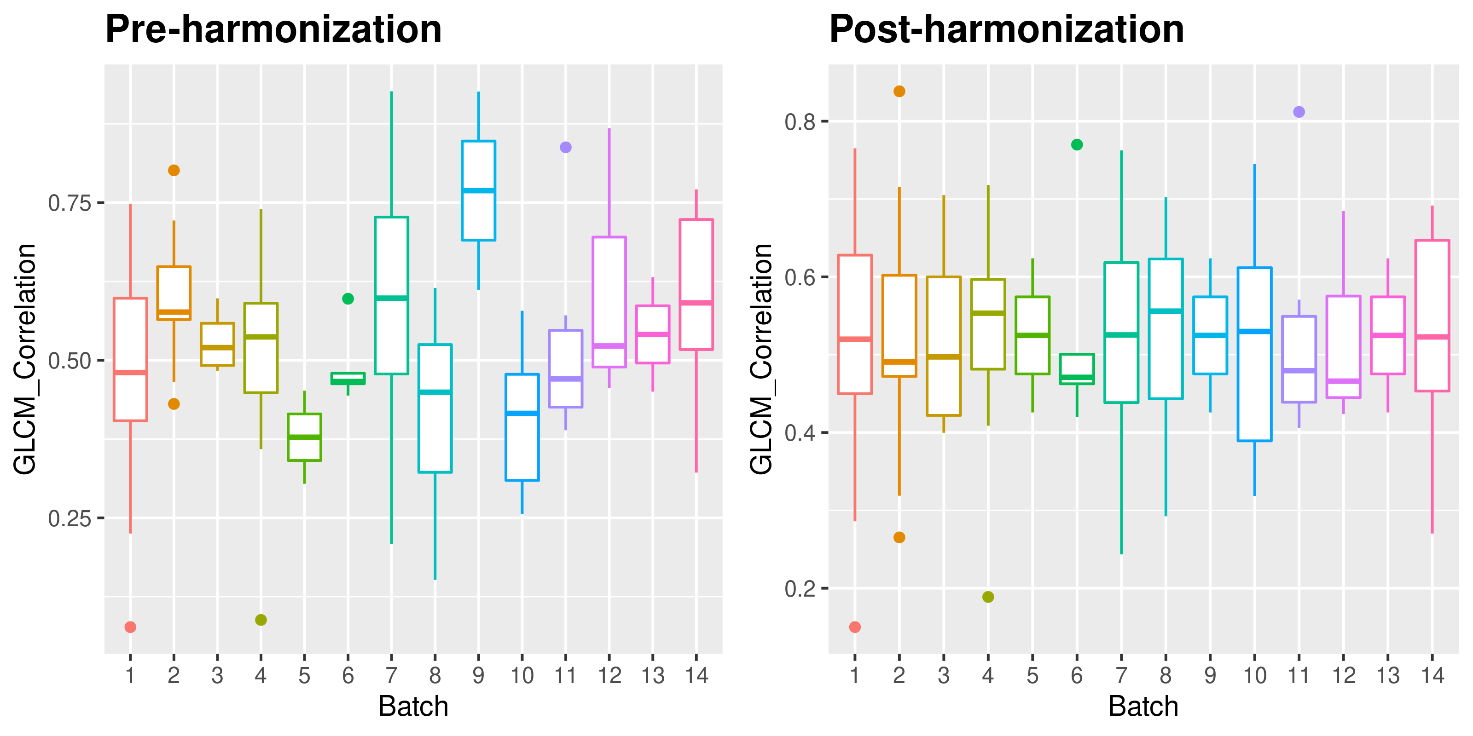


**Figure S2B**
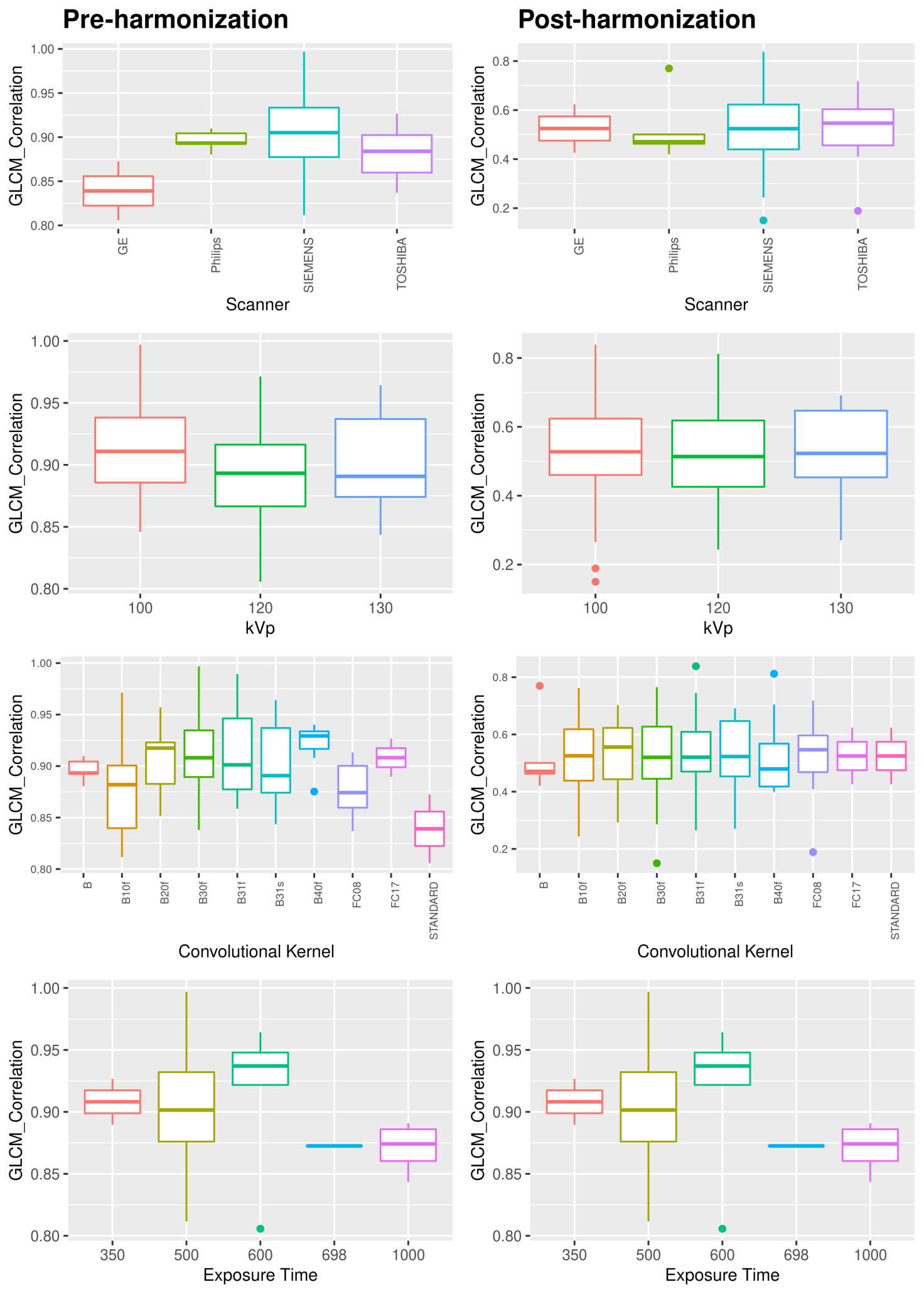


**Figure S3A**
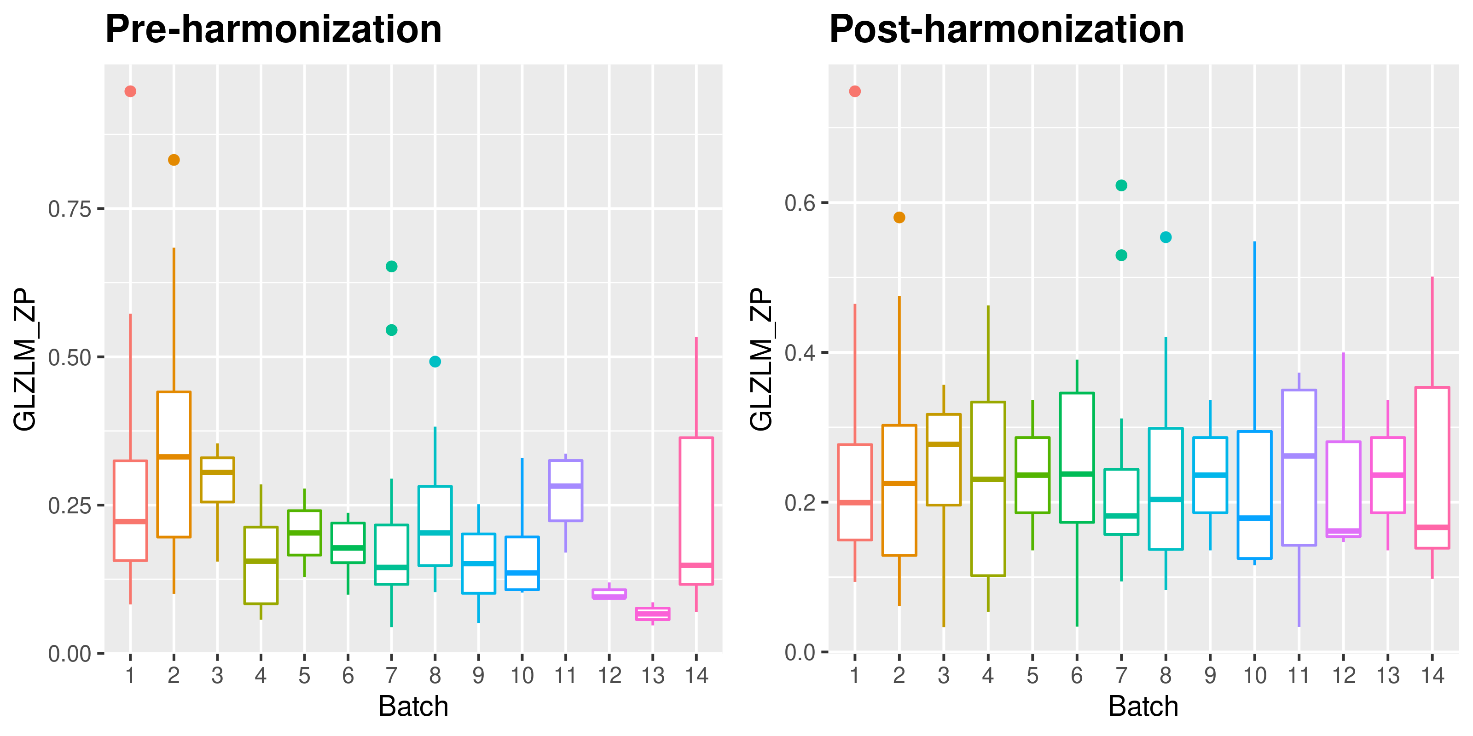


**Figure S3B**
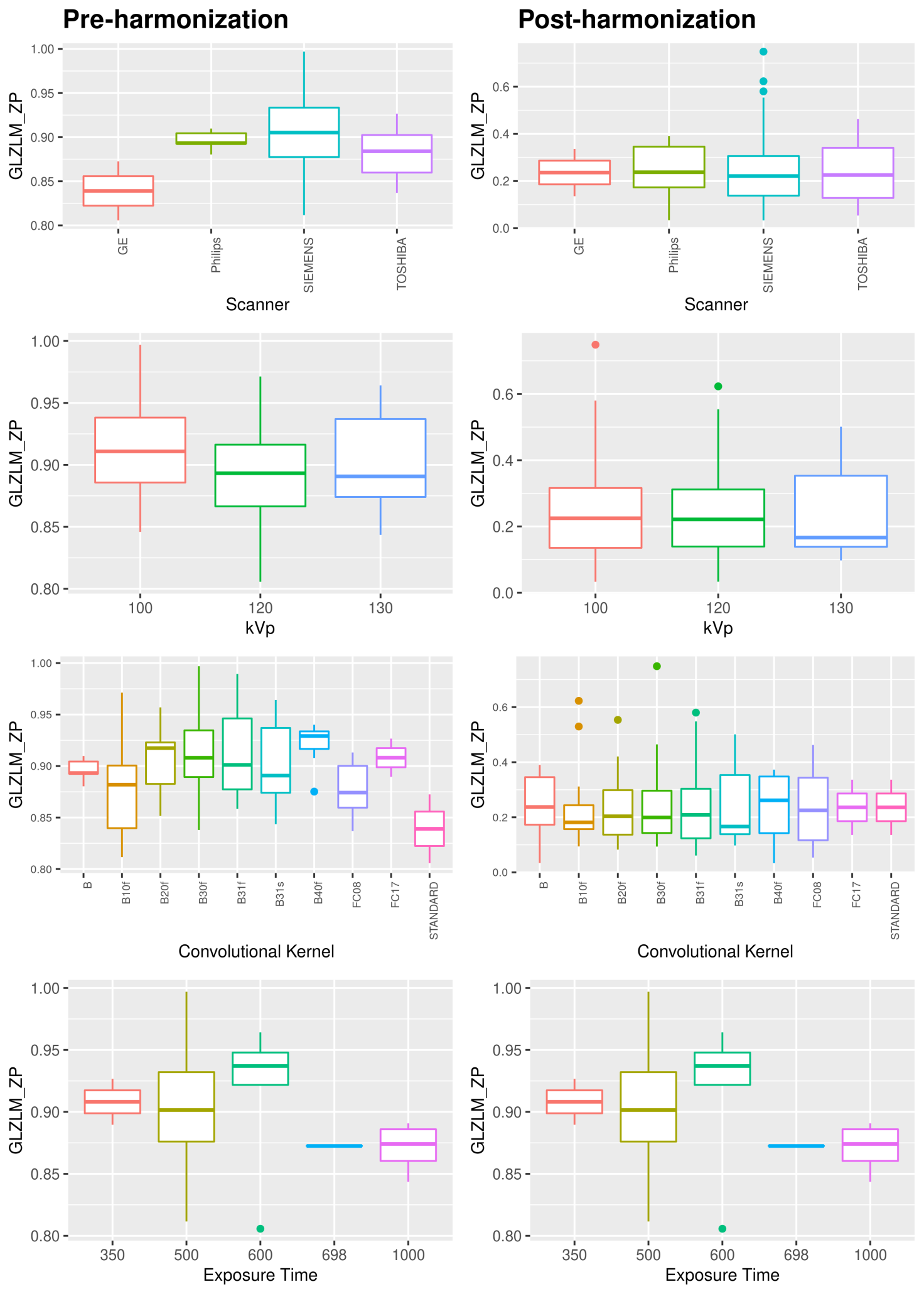


**Figure S4A**
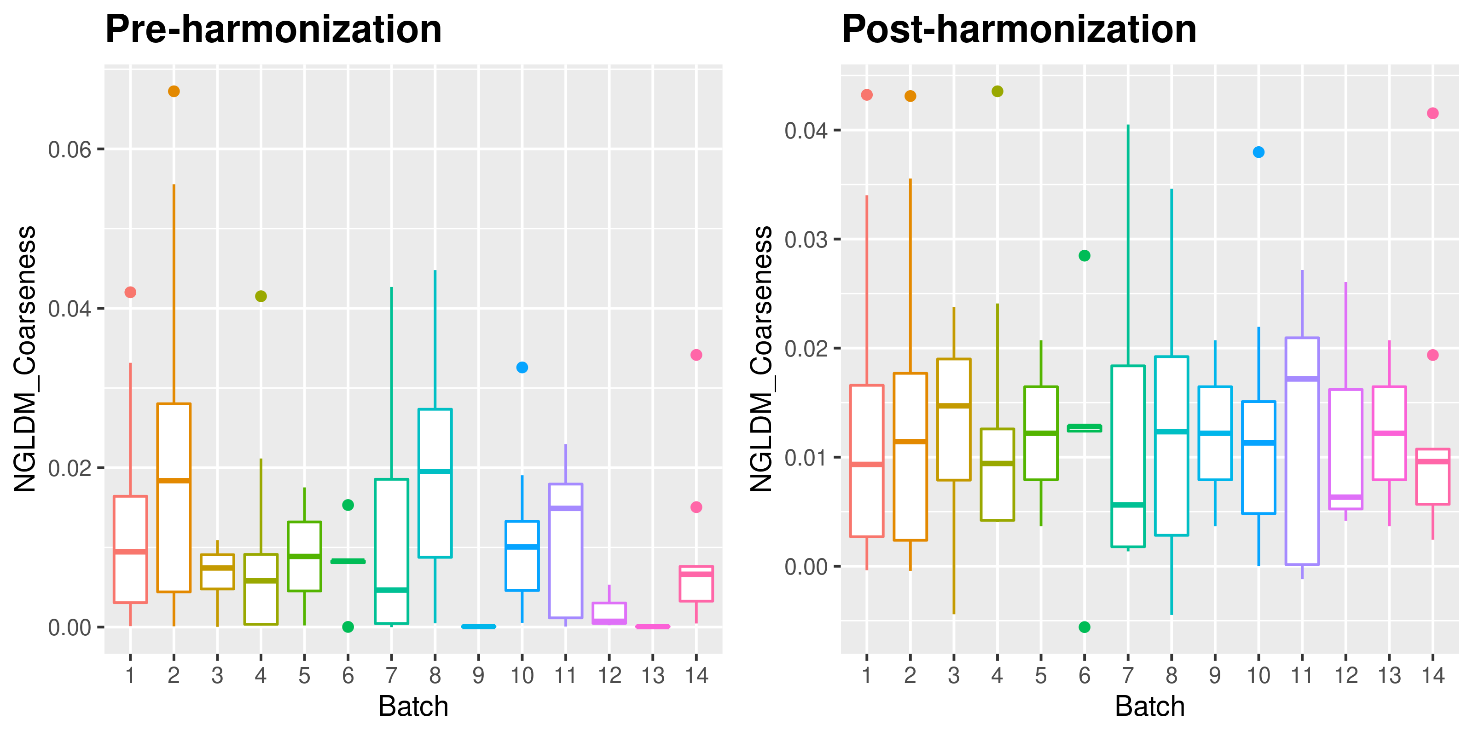


**Figure S4B**
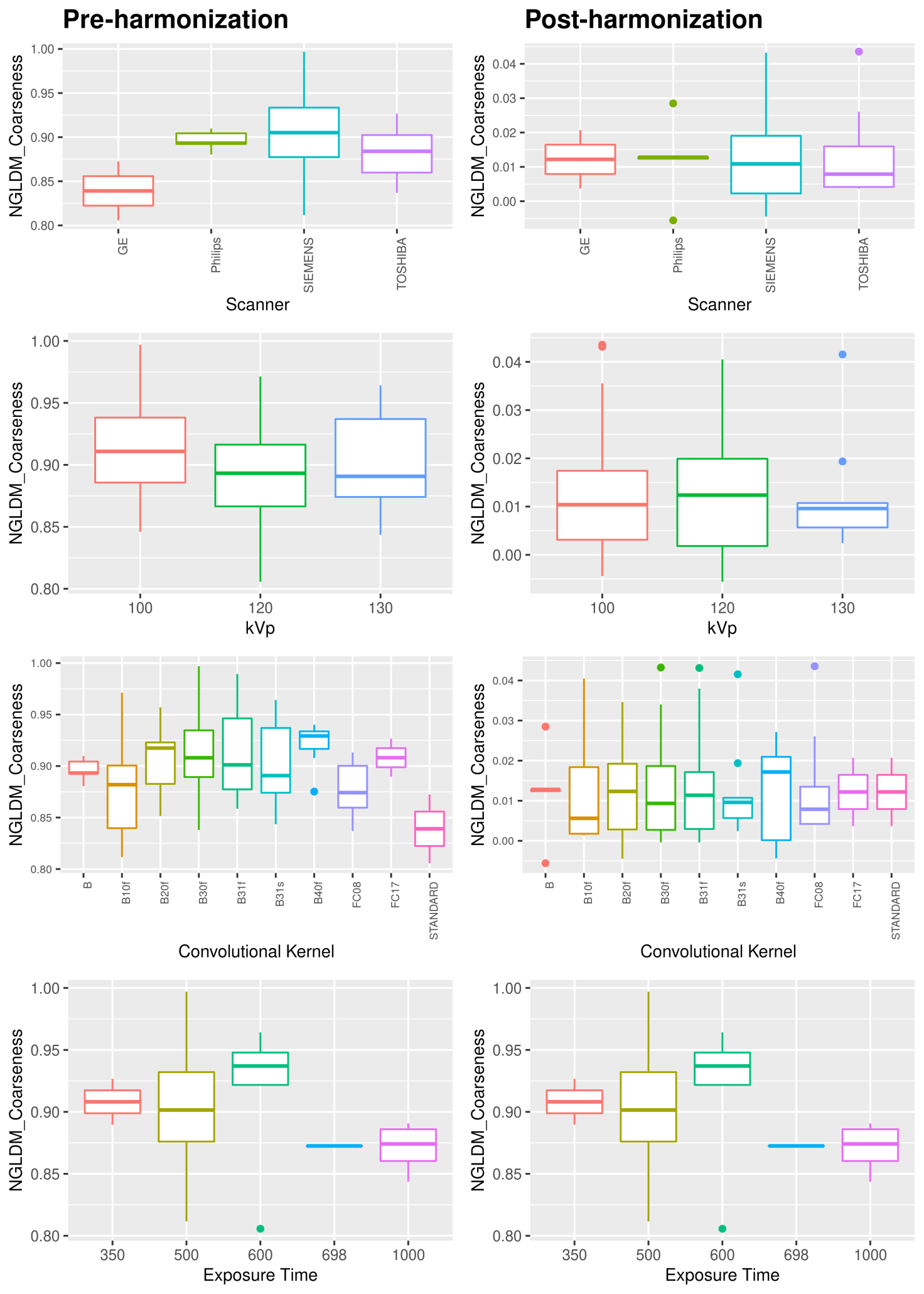


**Figure S5A**
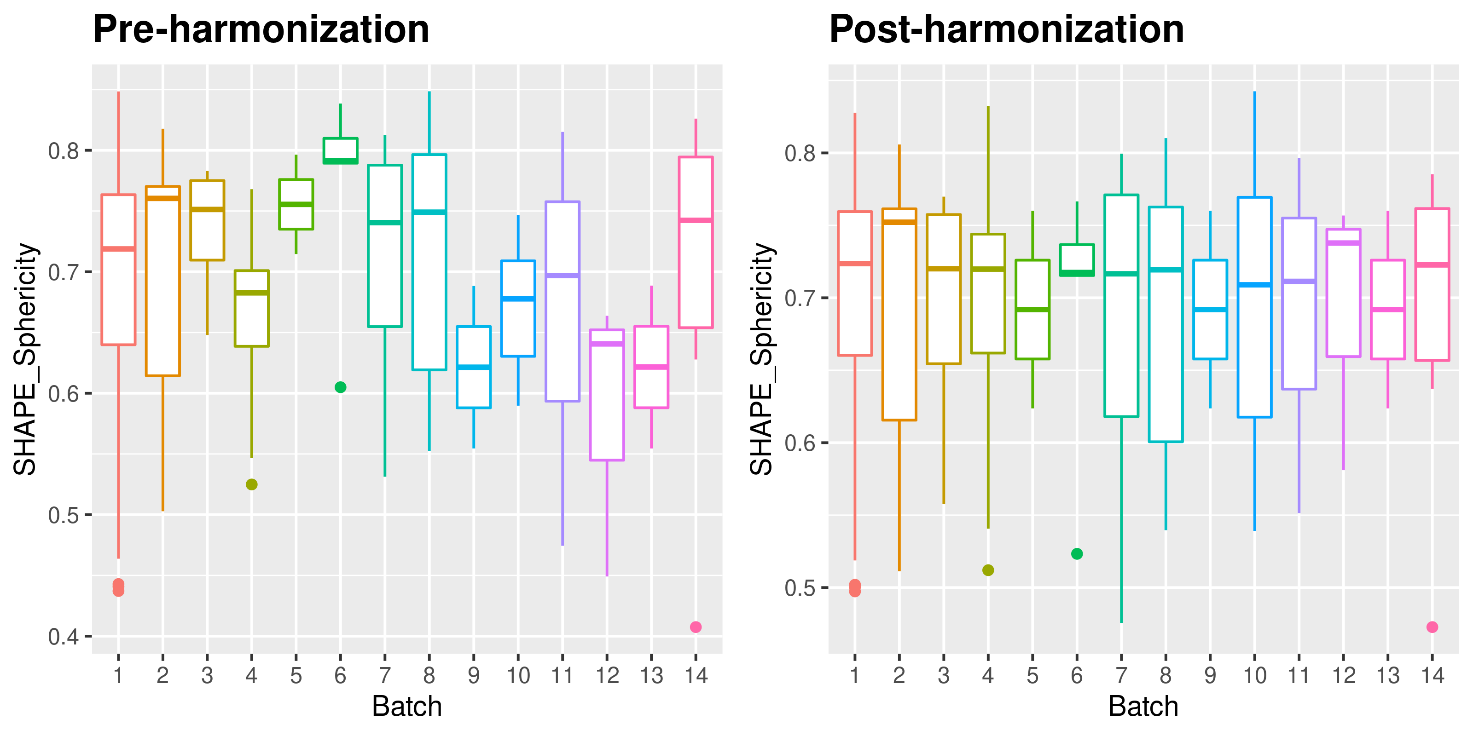


**Figure S5B**
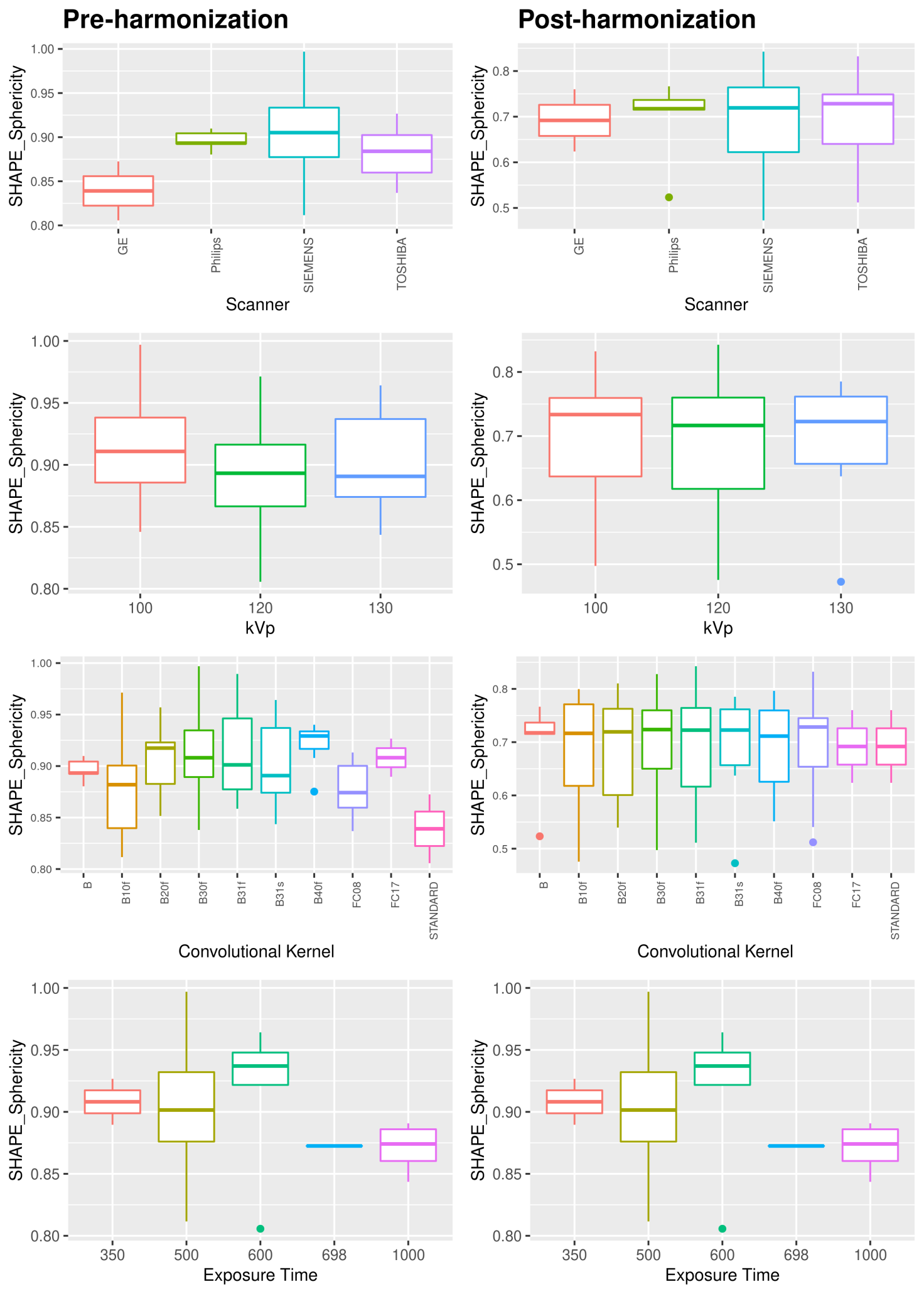


**Figure S6A**
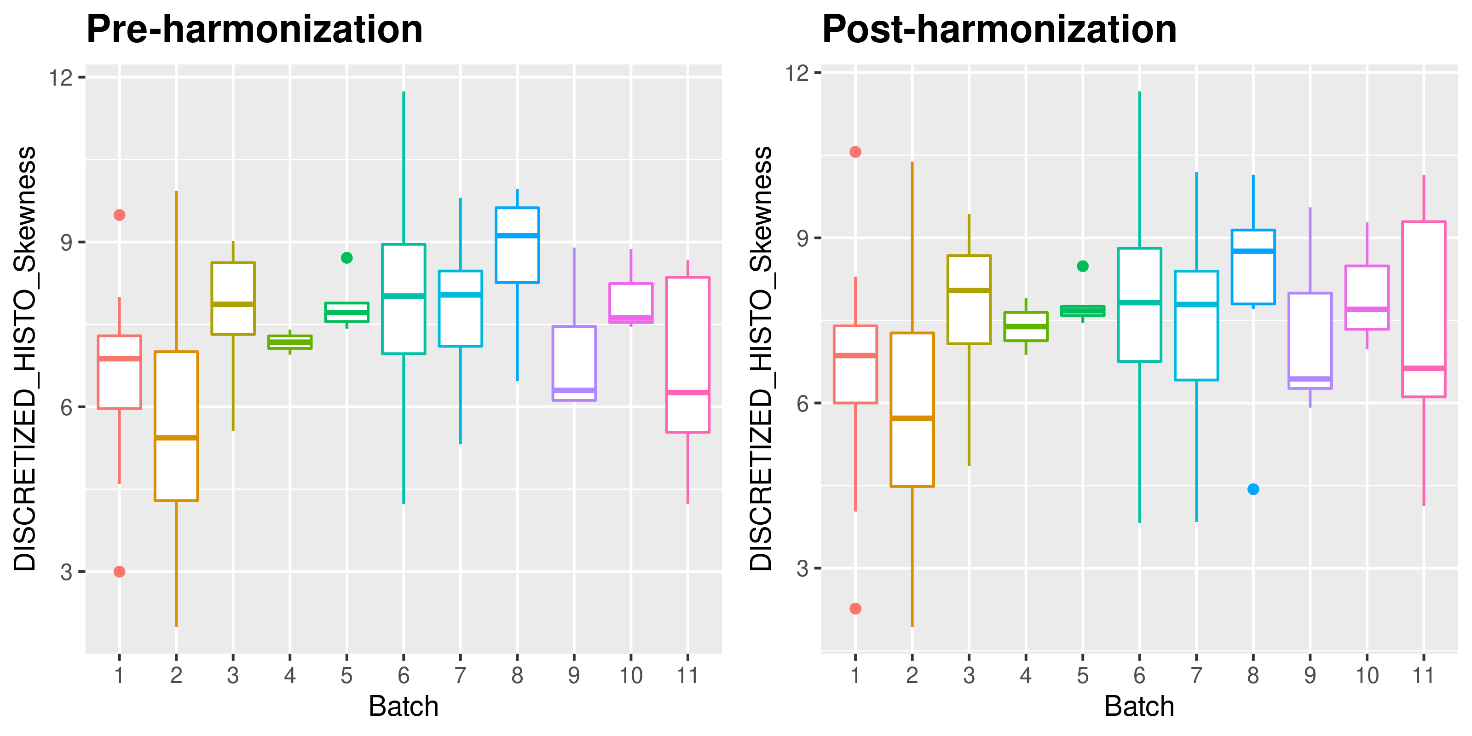


**Figure S6B**
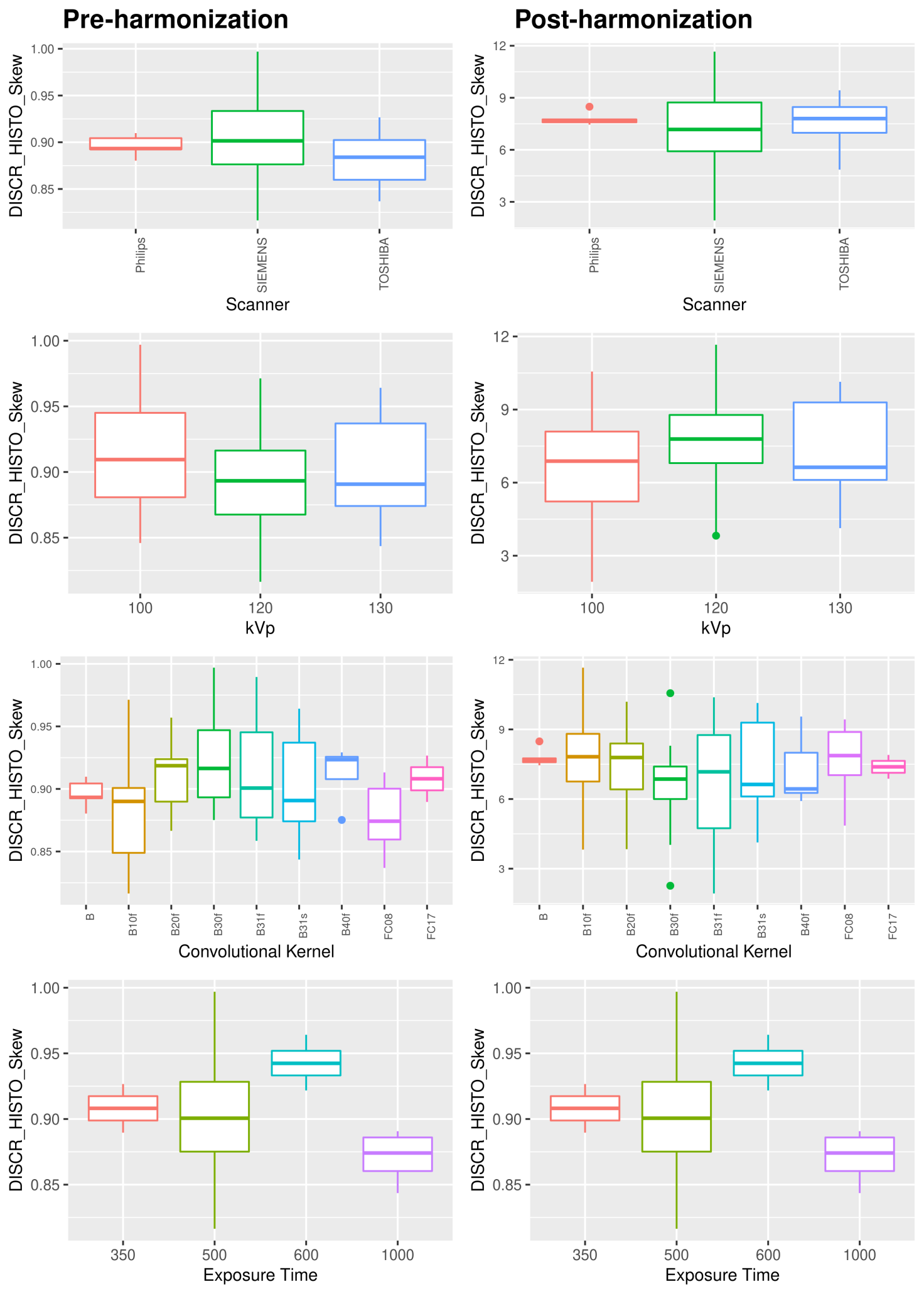


**Figure S7A**
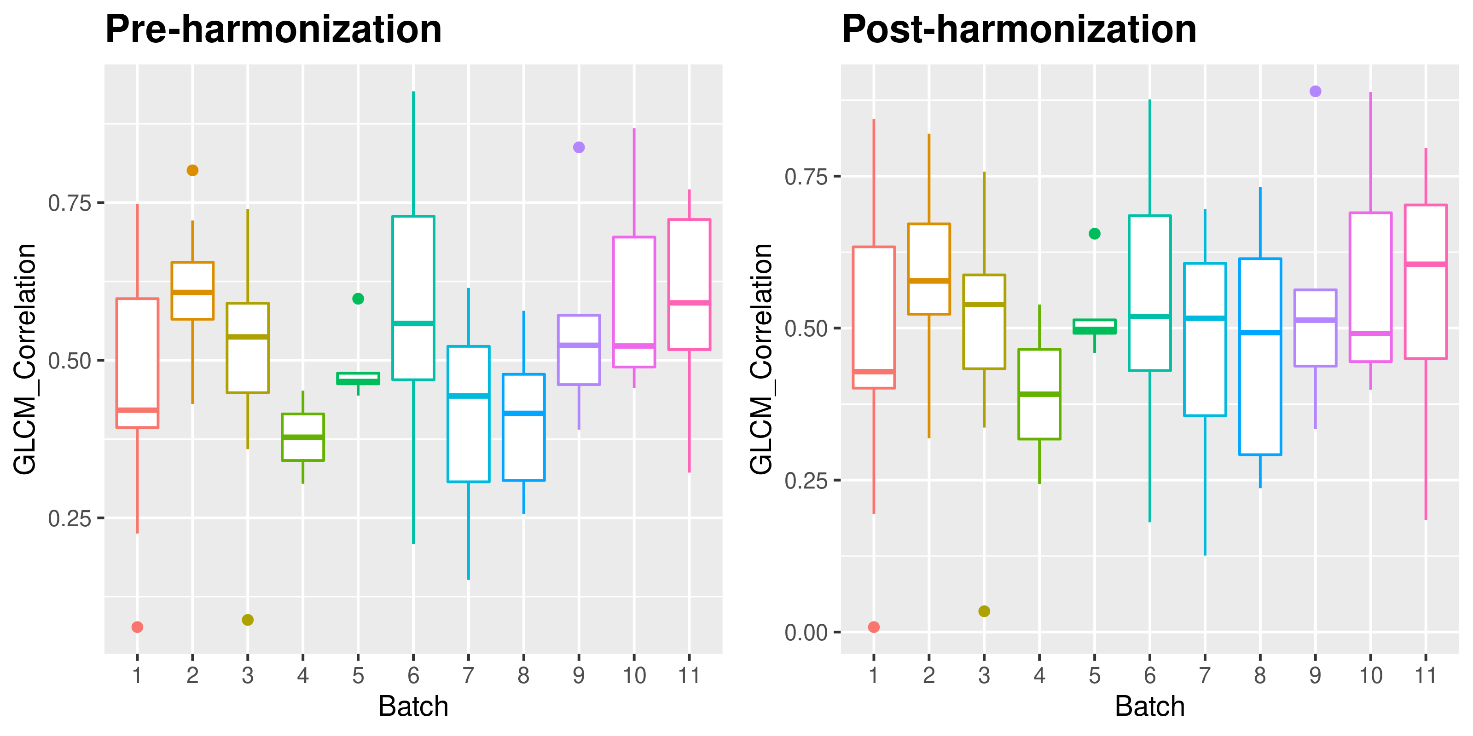


**Figure S7B**
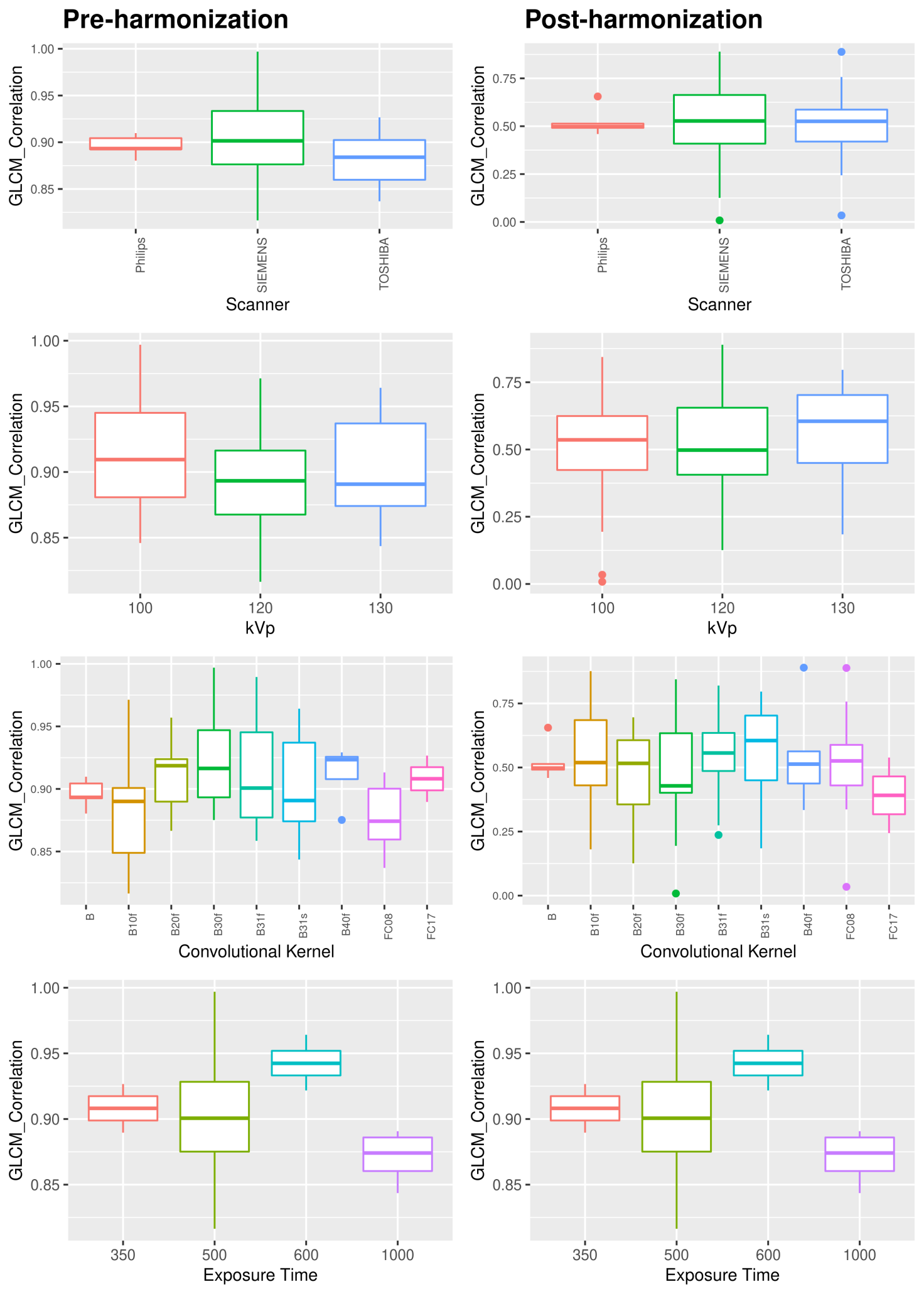


**Figure S8A**
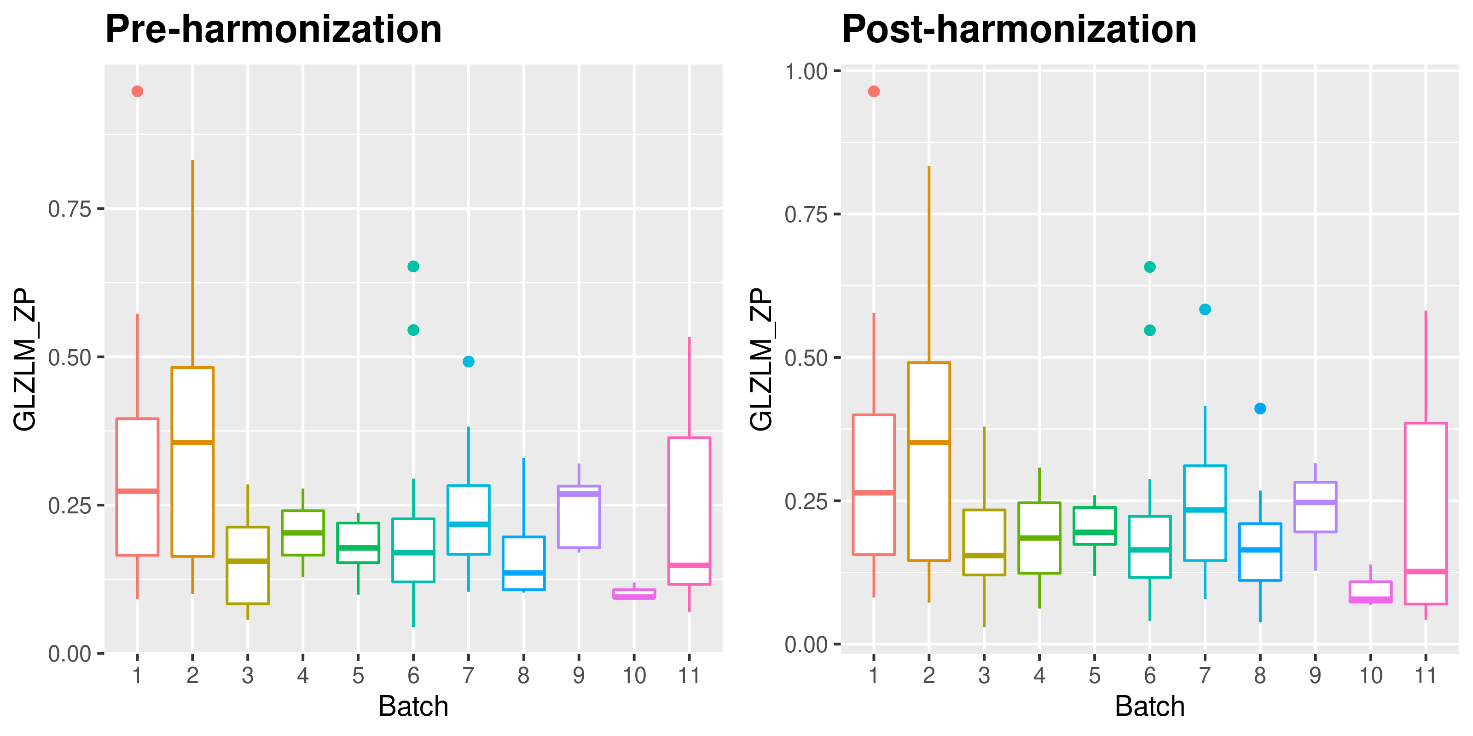


**Figure S8B**
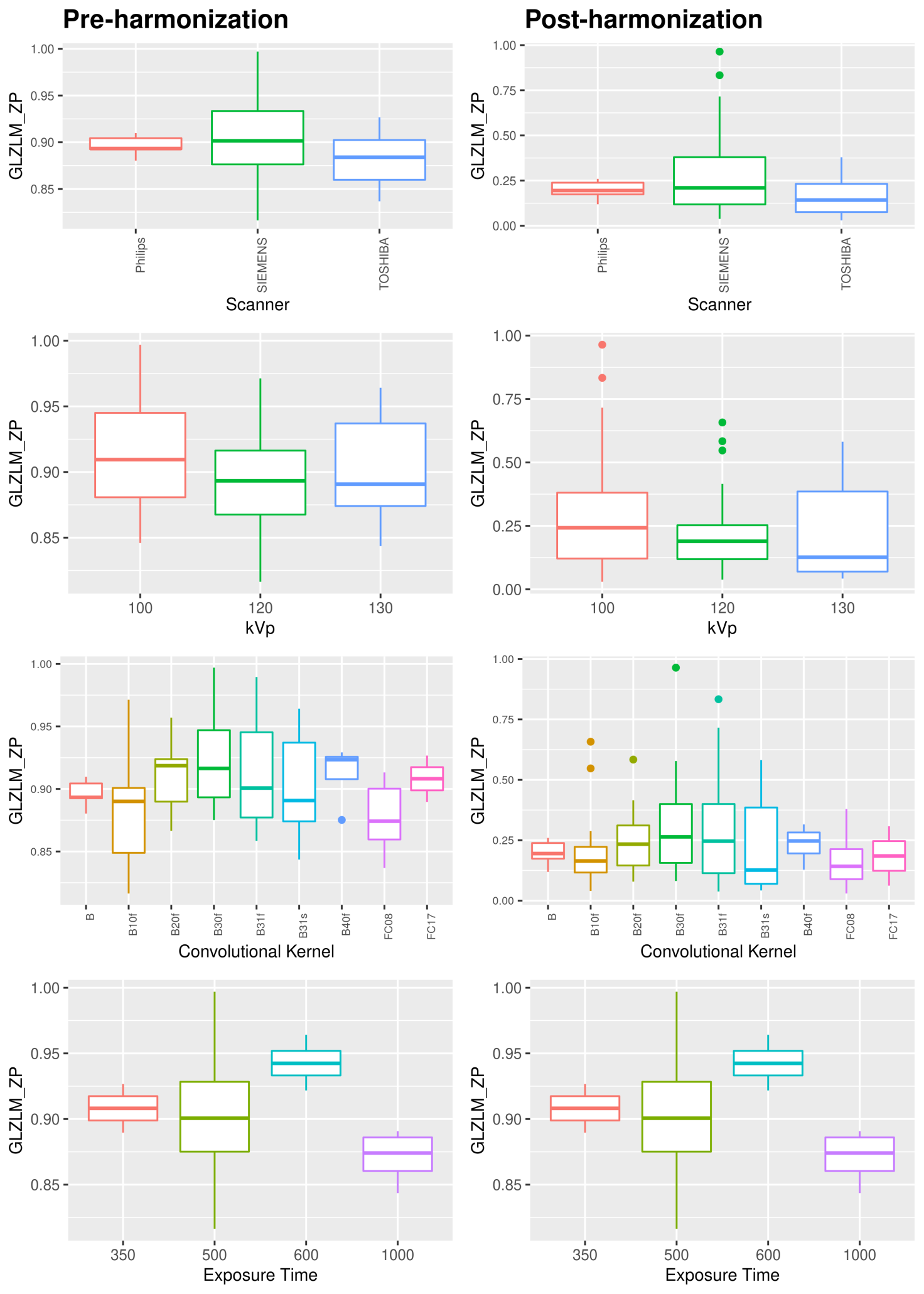


**Figure S9A**
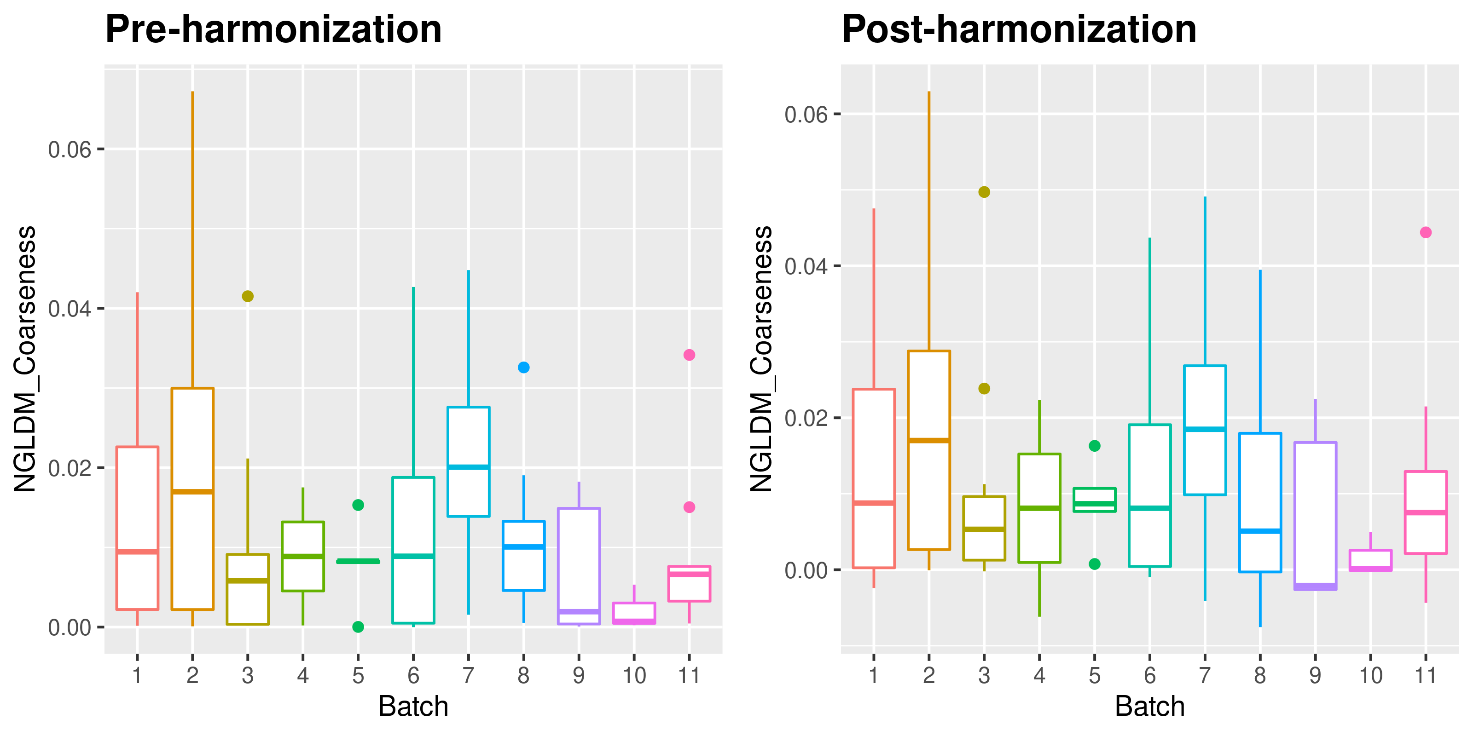


**Figure S9B**
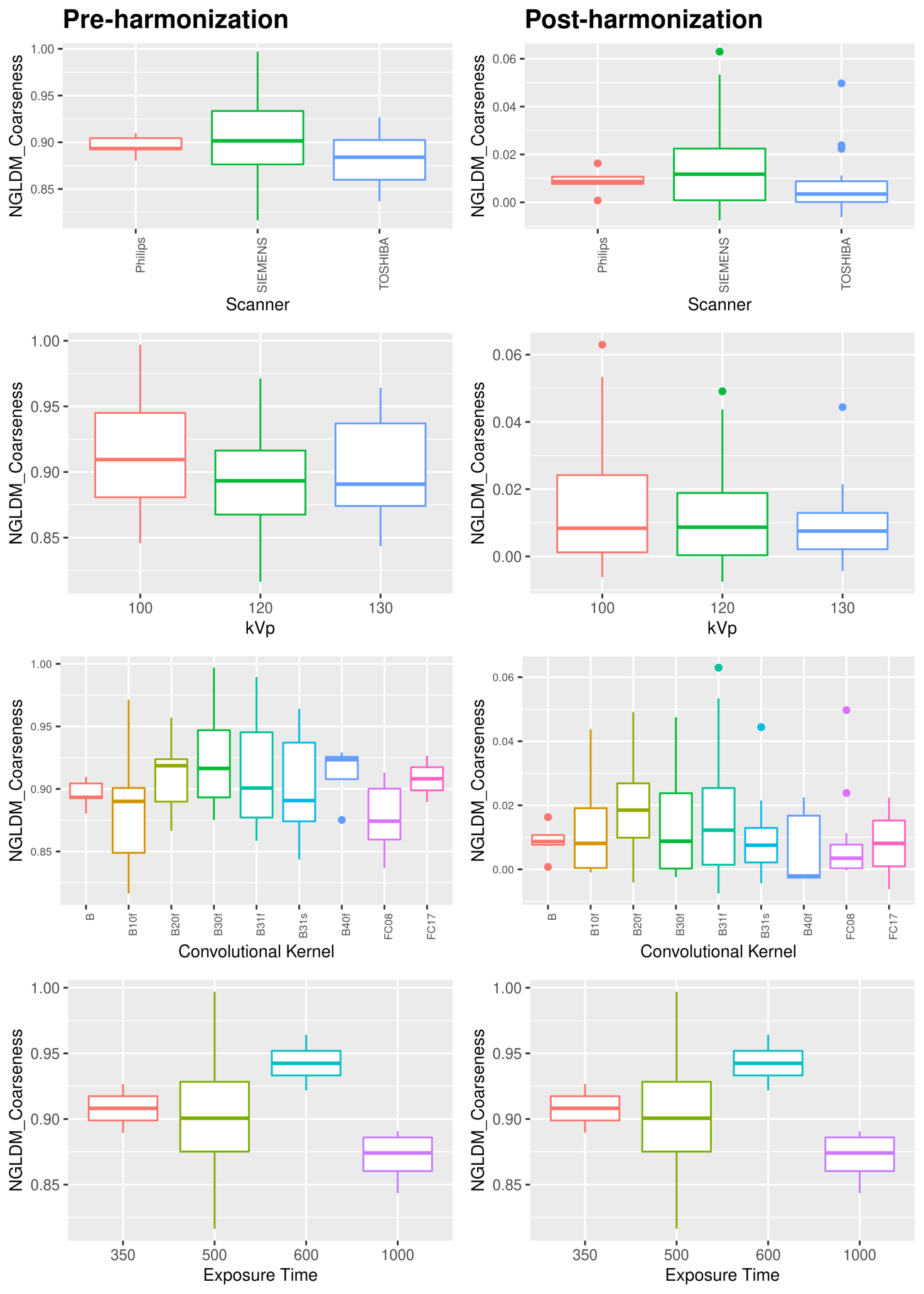


**Figure S10A**
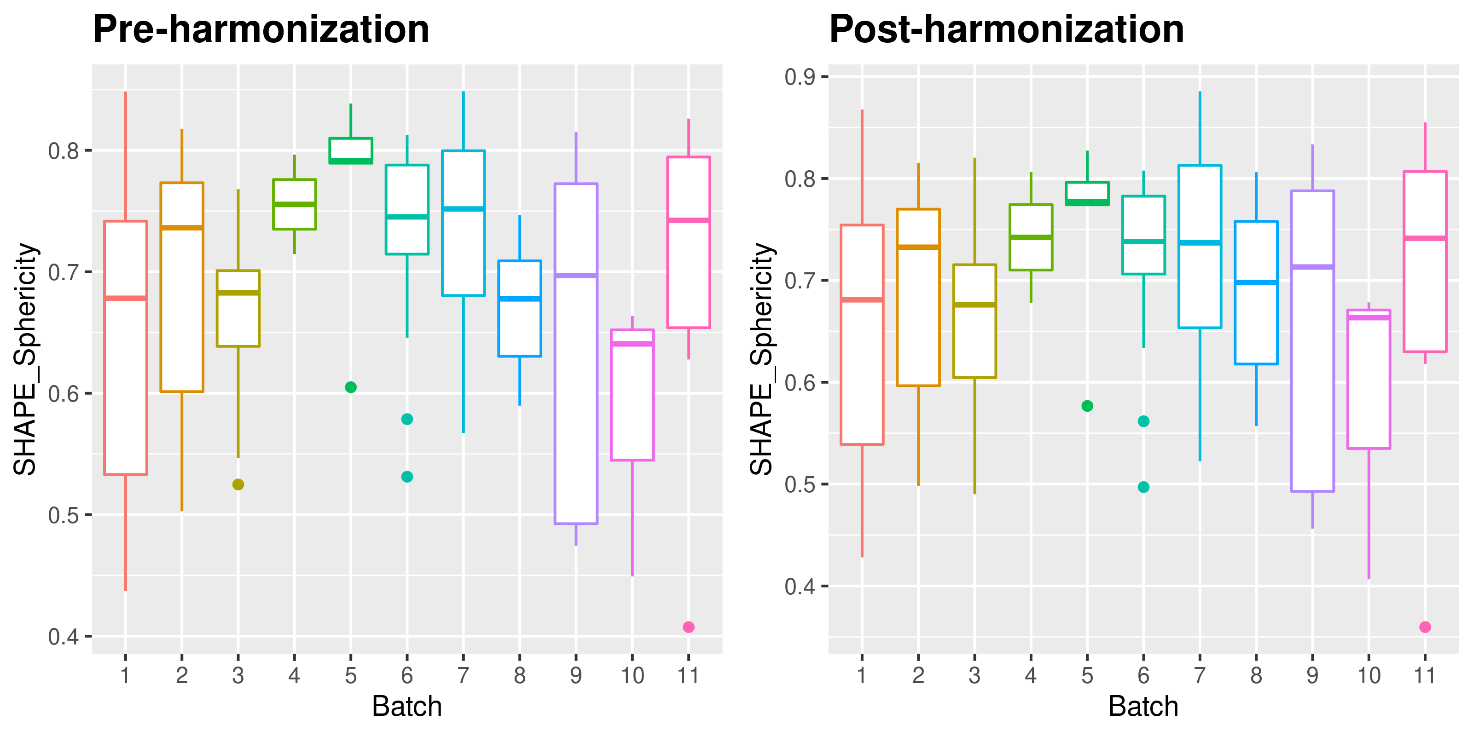


**Figure S10B**
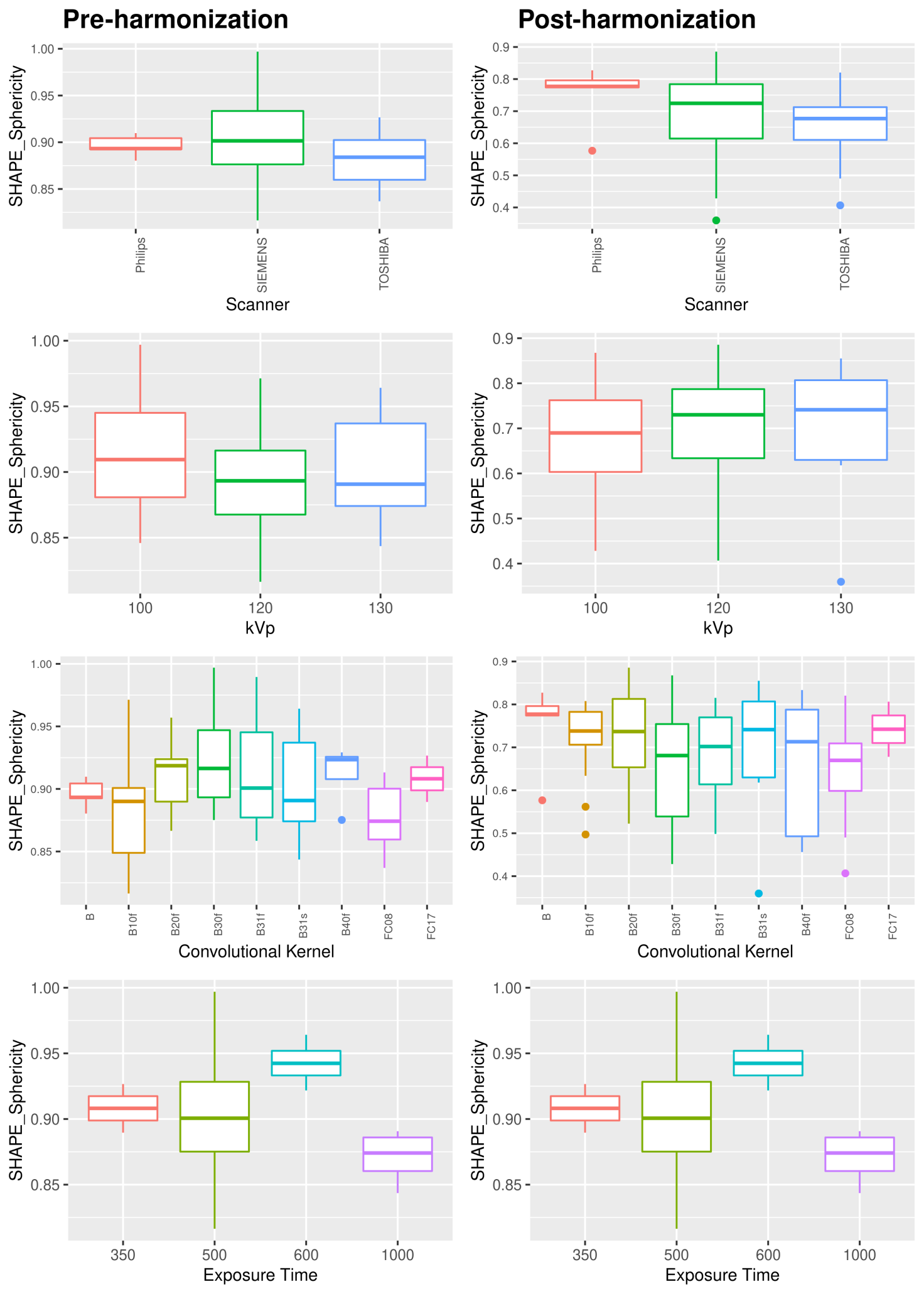


**Appendix A**

The ComBat harmonization technique belongs to the location and scale (L/S) adjustment methods which aim to eliminate the effects of batches by standardizing the means (location) and variances (scale) of each feature across batches.

This method assumes that the value of the feature k for the sample j from the batch i, represented by $Y_{i,j,k}$ can be written as:

$$Y_{i,j,k}=\alpha_{k}+\boldsymbol{X}\boldsymbol{\beta}_{k}+\gamma_{i,k}+\delta_{i,k}\varepsilon_{i,j,k}$$

where $\alpha_{k}$ is the average value of the feature $Y_{i,j,k}$, $\boldsymbol{X}$ is the design matrix of the biological covariates, $\boldsymbol{\beta}_{k}$ is the coefficient vector for the design matrix, $\gamma_{i.k}$ is the additive batch effect, $\delta_{i,k}$ is the multiplicative batch effect and $\varepsilon_{i,k}$ is the error of assuming a Normal distribution with an expected value of 0. The harmonized value of the feature called $Y_{i,j,k}^{ComBat}$is given by:

$$Y_{i,j,k}^{ComBat}=\frac{Y_{i,j,k}-\hat{\alpha_{k}}-\boldsymbol{X}\hat{\boldsymbol{\beta}_{k}}-\hat{\gamma_{i.k}}}{\hat{\delta_{i,k}}}+\hat{\alpha_{k}}+\boldsymbol{X}\hat{\boldsymbol{\beta}_{k}}$$

Where $\hat{\alpha_{k}}$, $\hat{\boldsymbol{\beta}_{k}}$, $\hat{\gamma_{i.k}}$ and $\hat{\delta_{i,k}}$ are the estimators of $\alpha_{k}$, $\boldsymbol{\beta}_{k}$, $\gamma_{i.k}$ and $\delta_{i,k}$ found through a maximum likelihood method using the set of available observations of the feature *k* for each batch *i*.

We chose the non-parametric setting of the model to avoid assumptions on the underlying probability distributions of the features and the parameters and we excluded the Bayesian formulation. Moreover, we did not include biological covariates (i.e., $\boldsymbol{X}=0$), because the cohort of patients enrolled for the *BLINDED* study is very homogeneous, and we do not have a significant biological feature which we would like to preserve from harmonization. By selecting these options, the algorithm computes a location- and-scale correction transformation for each feature separately, i.e., it adjusts the means and the variances of the distributions to reduce heterogeneity.

We used the ComBat tool proposed in [21] and adapted to multi-site imaging data in [19], publicly available at https://github.com/Jfortin1/ComBatHarmonization, using the R software.

Moreover, we used a development of the ComBat tool, named longComBat, as defined in [20]. In longComBat, the original ComBat algorithm was adapted to longitudinal data when the independence requirement between statistical units was not satisfied. As far as we know, the application of this technique to radiomic features has not been studied yet. The longComBat method assumes that the value of the features k of the batch i for the observation j at time t, $Y_{i,j,k}$, can be modeled by:

$$Y_{i,j,k}\left( t \right)=\alpha_{k}+\boldsymbol{X}_{\boldsymbol{j}}^{\boldsymbol{T}}\left( t \right)\boldsymbol{\beta}_{\boldsymbol{k}}+\gamma_{i.k}+\eta_{i.k}+\delta_{i,k}\varepsilon_{i,j,k}\left( t \right)$$

where *t* is the time which can be a categorical or continuous variable, $\alpha_{k}$ is the average value of $Y_{i,j,k}\left( t \right)$ at the baseline, $\boldsymbol{X}_{j}\left( t \right)$ is the the design matrix of the time-dependent covariates (in our case only the time variable), $\boldsymbol{\beta}_{\boldsymbol{k}}$ is the coefficient vector for the design matrix, $\gamma_{i.k}$ and $\delta_{i,k}$ are the additive batch effect and the multiplicative batch effect respectively, $\eta_{i.k}$ is a subject-specific random intercept and $\varepsilon_{i,j,k}\left( t \right)$ is the error term. It is assumed that both the terms $\eta_{i.k}$ and $\varepsilon_{i,j,k}\left( t \right)$ follow a normal distribution with an expected value of 0. The

longComBat harmonized feature value $Y_{i,j,k}^{longComBat}\left( t \right)$ is:

$$Y_{i,j,k}^{longComBat}\left( t \right)=\frac{Y_{i,j,k}\left( t \right)-\hat{\alpha_{k}}-\boldsymbol{X}_{\boldsymbol{j}}^{\boldsymbol{T}}\left( t \right)\hat{\boldsymbol{\beta}_{k}}-\hat{\gamma_{i,k}}-\hat{\eta_{i,k}}}{\hat{\delta_{i,k}}}+\hat{\alpha_{k}}+\boldsymbol{X}_{\boldsymbol{j}}^{\boldsymbol{T}}\left( t \right)\hat{\boldsymbol{\beta}_{k}}$$

where the parameter estimates $\hat{\alpha_{k}}$, $\hat{\boldsymbol{\beta}_{k}}$, $\hat{\gamma_{i,k}}$*,* $\hat{\eta_{i,k}}$ and $\hat{\delta_{i,k}}$ for longComBat are found in a similar way to standard ComBat.

Unlike the cross-sectional ComBat tool, the longitudinal version is not yet provided with the non-parametric adjustment nor the possibility to exclude the Bayesian setting. We use longCombat algorithm publicly available at https://github.com/jcbeer/longCombat, using the R software.
